# Clinical and neuroimaging features, and associations with early post-stroke cognition in patients with intracerebral hemorrhage in Taiwan

**DOI:** 10.1371/journal.pone.0358545

**Published:** 2026-09-21

**Authors:** Chung-Fen Tsai, Sung-Chun Tang, Ya-Hui Wang, Chih-Hao Chen, Kristiina Rannikmäe, Ping-Keung Yip, Jiann-Shing Jeng

**Affiliations:** 1 Department of Neurology, Cardinal Tien Hospital and Fu Jen Catholic University, New Taipei City, Taiwan; 2 Stroke Center and Department of Neurology, National Taiwan University Hospital, Taipei, Taiwan; 3 Medical Research Center, Cardinal Tien Hospital, New Taipei City, Taiwan; 4 Centre for Clinical Brain Sciences, University of Edinburgh, Edinburgh, United Kingdom; University of Louisville School of Medicine, UNITED STATES OF AMERICA

## Abstract

**Background:**

East Asians have a higher incidence rate of intracerebral hemorrhage (ICH). As clinical and neuroimaging characteristics, genetic and vascular risk factors, and prognosis might vary between populations, we aimed to make comparisons between different ICH locations and etiologies, and to investigate the factors associated with early post-stroke cognition in Chinese patients in Taiwan.

**Methods:**

We prospectively recruited acute spontaneous Chinese ICH patients from two large Hospitals in Taiwan. For included patients, we compared major vascular risk factors, APOE genotypes, clinical and neuroimaging features, and early post-stroke cognition in patients with lobar versus non-lobar ICH and in cerebral amyloid angiopathy (CAA) versus non-CAA group. Furthermore, we used generalized linear models to investigate the associated factors with early global cognitive performance after ICH.

**Results:**

In 202 included acute spontaneous ICH patients, 65% were men. 80% were non-lobar ICH, and 20% were lobar ICH. Lobar ICH patients generally had an older age, worse conscious level, a higher 1-month case fatality, a larger ICH volume, more lobar CMBs and cortical superficial siderosis, and worse early post-stroke cognitive function, while non-lobar patients had a higher proportion of hypertension (98% vs 85%, p < 0.001). Similar findings were noted when comparing patients of CAA group with non-CAA groups. Generalized linear regression models revealed that age, education, initial conscious level, stroke severity, ICH volume and lobar CMB burden were associated with early post-stroke cognition in ICH patients.

**Conclusion:**

Our study demonstrated the differences in clinical and imaging features, major risk factors, and early post-stroke cognitive function between different locations and etiologies in Chinese ICH patients in Taiwan. Age, education, stroke severity, ICH volume and lobar CMB burden were the associated factors for early global cognitive performance after ICH.

## Introduction

Stroke is a major global disease and the third cause of death worldwide [1]. While the age-adjusted incidence of stroke has decreased in many Western countries in recent years, the annual number of strokes has increased in Asia [1]. Among pathological types of strokes, ICH is mostly catastrophic with high mortality and disability rates [2]. The incidence rate and proportion of ICH are significantly higher in East Asians, including Chinese populations, leading to a heavy stroke burden [2,3].

Some studies report that risk factors differ between lobar and non-lobar (deep) ICH subtypes when ICH is categorized anatomically [4]. Hypertension poses the greatest risk for deep ICH, while cerebral amyloid angiopathy (CAA) is the major risk factors for lobar ICH, which is possibly associated with apolipoprotein E (APOE) genotypes ε4 [4,5]. However, most studies are based on Western stroke patients, with limited data on Chinese patients. Recently, advances of novel neuroimaging markers have provided more understanding of ICH such as cerebral microbleeds (CMBs). They are small hypointensities on susceptibility weighted imaging (SWI) or T2*-weighted Magnetic Resonance Imaging (MRI), corresponding to recent or old microhemorrhage in histopathological examinations [6,7]. CMBs are prevalent in ICH and different distributions and loading of CMBs may relate to various etiologies, risk of recurrent stroke, post-stroke cognitive impairment and outcome [8].

Given the higher incidence and proportion of ICH in Chinese populations and the possibly different risk factors and impacts of stroke between populations, we designed the study to evaluate whether clinical and neuroimaging features, major genetic and vascular risk factors varied in different ICH locations and etiologies in Chinese ICH patients in Taiwan. In addition, we investigated the associations of clinical, genetic, and neuroimaging factors with early post-stroke cognitive function in these ICH patients.

## Methods

We prospectively recruited acute spontaneous ICH adult patients within one month of stroke onset from National Taiwan University and Cardinal Tien Hospitals in Taiwan from Aug 2018 to July 2022. A neurologist assessed each patient, recorded clinical information, and arranged brain computed tomography (CT) on the day upon arrival. We reviewed medical and drug history, checked major vascular risk factors and APOE genotypes, conducted brain MRI and angiography including SWI (within 1 week after admission), and performed cognitive evaluation between the first and second month after stroke if possible. This study protocol was reviewed and approved by National Taiwan University and Cardinal Tien Hospital Institutional Review Boards (CTH-106-2-1-079). Written informed consent was obtained from all the participants or their families.

### Diagnosis of spontaneous intracerebral hemorrhage and classification

Diagnosis of ICH was based on clinical features: acute headache, vomiting, conscious change, seizure, or focal neurological deficits, combined with blood within brain parenchyma on brain CT or MRI. ICH was classified anatomically as lobar (cortical-subcortical areas) and non-lobar (basal ganglia, thalamus, periventricular white matter, cerebellum and brainstem) locations. Also, we conducted etiological classification based on SMASH-U and divided patients into CAA and non-CAA groups according to Boston criteria version 2.0 [9,10]. Major risk factors included hypertension, diabetes, atrial fibrillation, ischemic heart disease, hypercholesterolemia, hypertriglyceridemia, current or former smoking, habitual alcohol drinking (more than once per week), history of previous stroke, pre-ICH antiplatelet or anti-coagulant use, and family history of stroke. Patients were excluded if they had traumatic ICH, purely subdural/epidural/subarachnoid/intraventricular hemorrhage, post-infarct hemorrhagic transformation, tumor bleeding or non-cerebrovascular cause, no genetic study or unavailable informed consent.

### Genetic study

For the genetic study, we collected blood from all participants to extract DNA using standard techniques. Sanger sequencing was used for genotyping the three alleles APOE ε2/ε3/ε4 and allelic frequencies were calculated by gene counting. We classified the APOE genotype as APOE ε4 carriers if participants had at least one ε4 allele (ɛ2/ɛ4, ɛ3/ɛ4, ɛ4/ɛ4) and non-APOE ɛ4 carriers (ɛ2/ɛ2, ɛ2/ɛ3, ɛ3/ɛ3). Genotypers were blind to clinical information and image findings.

### Cerebral microbleeds and other novel neuroimaging markers on brain MRI

We assessed all included patients with standard brain MRI (3.0 T) imaging, MR angiography, and SWI imaging. We used Microbleed Anatomical Rating Scale to assess and localize CMBs, classifying as lobar, non-lobar, and mixed (presence of CMBs in both lobar and deep areas), and counted the number of CMBs [11]. We also evaluated the volume of ICH, presence of cortical superficial siderosis (cSS), lacunes, white matter hyperintensities (WMH), perivascular spaces (PVS), and brain atrophy. cSS and lacunes were recorded as present if at least 1 lesion was indentified on MRI. WMH and PVS were recorded if moderate to severe (≥ grade 2 WMH, PVS > 10 visible PVS) by visual scoring system [12,13]. Image reading was performed independently by two trained observers, blinded to genetic tests.

### Cognitive evaluation

For patients with cooperative status, we assessed the global cognitive function by the Taiwanese version of Mini-Mental State Examination (MMSE) and Montreal Cognitive Assessment (MoCA) between the first and second month after ICH (after acute phase) [14,15]. We also compared the proportion of cognitive impairment between groups, which was defined as: < 24 based on MMSE score for patients with education of 6 years or more and <14 for less than 6 years; < 24 on MoCA [14,15].

### Statistical analysis

We used Pearson’s chi-square or Fisher’s exact test to compare the proportions of major vascular risk factors, APOE ɛ4 carrier, neuroimaging findings and post-stroke cognitive impairment in patients with lobar ICH versus non-lobar ICH, and in CAA versus non-CAA group. Student’s t test was applied to compare the continuous variables between groups. In addition, we made comparisons between patients with APOEɛ4 and non-APOEɛ4.

To investigate the associations with early global cognitive function in ICH patients, we included clinical and neuroimaging variables of clinical significance and literature information into generalized linear regression models for multivariable analysis and used stepwise selection method. Model I adjusted for clinical important variables like age, sex, education year, National Institutes of Health Stroke Scale (NIHSS), Glasgow Coma Scale (GCS), ICH location, major vascular risk factors (hypertension, diabetes, atrial fibrillation, ischemic heart disease, hypercholesterolemia, hypertriglyceridemia, smoking, alcohol and previous stroke), CAA and APOE genotypes. Model II included both clinical and neuroimaging variables, such as ICH volume, CMBs and other neuroimaging markers on MRI. To test for multicollinearity in this study, we used variance inflation factors (VIF) and tolerance to examine these variables in the final multivariable linear regression models. For missing data, we adapted deletion methods (pairwise deletion), without imputation. Also, we performed sensitivity analysis, excluding 3 patients with a diagnosis of dementia before ICH.

Furthermore, we conducted subgroup analysis to assess the impact of lobar or non-lobar CMB loading (divided into quartiles) on post-stroke cognition. Statistical tests were two-sided, with p values <0.05 considered significant. Analyses were performed using SAS version 9.4 (SAS Institute Inc, Cary, NC, USA).

## Results

### Clinical characteristics and major vascular and genetic risk factors

Totally we recruited 202 ICH patients, with a mean age of 60.5 years (± 12.4); 65% were men. There were 41 lobar ICH (20%) and 161 non-lobar ICH patients (80%). All patients had brain CT and 92% had brain MRI/MRA including SWI. The study flow chart was in Supporting information (S1 Fig). Patients with lobar ICH were older (mean age 66.4 vs 58.8 years, p = 0.001), had worse initial GCS (13.0 vs 14.0, p = 0.011) and a higher 1-month case fatality (7% vs 0%, p = 0.008) than those with non-lobar ICH, while the distribution of sex, mean NIHSS and ICH score did not differ significantly (Table 1).

**Table 1:** Comparisons of clinical, genetic, neuroimaging features, and post-stroke cognition between lobar and non-lobar ICH patients.

ICH subtypes (N = 202)	Lobar ICH (N = 41) (20%)	Non-lobar ICH (N = 161) (80%)	P value
Mean age (year)	66.4 (± 12.6)	58.8(± 11.8)	0.001*
Sex (male) (N) (%)	25 (61%)	106 (66%)	0.560
GCS	13.0 (± 3.2)	14.0 (± 2.1)	0.011*
NIHSS	9.4 (± 11.4)	8.8 (± 6.9)	0.683
ICH score	1.0 (**±** 1.1)	0.7 (**±** 0.9)	0.073
1M case fatality	3 (7%)	0 (0%)	0.008*
CAA	25 (61%)	0 (0%)	<0.001*
Hypertension(N) (%)	35 (85%)	158 (98%)	<0.001*
Diabetes (N) (%)	8 (22%)	40 (25%)	0.474
Atrial fibrillation (N) (%)	4 (10%)	7 (4%)	0.173
Ischemic heart(N) (%)	2 (5%)	18 (11%)	0.228
Hypercholesterolemia(N) (%)	9 (22%)	46 (29%)	0.355
Hypertriglyceridemia (N) (%)	1 (2%)	12 (7%)	0.243
Smoking (N) (%)	23 (56%)	83 (52%)	0.603
Alcohol (N) (%)	9 (22%)	38 (24%)	0.823
Previous stroke(N) (%)	10 (24%)	21 (13%)	0.072
Pre-ICH antiplatelet(N) (%)	7 (17%)	17 (11%)	0.250
Pre-ICH anticoagulation(N) (%)	2 (5%)	4 (2%)	0.420
Family history of stroke (N) (%)	10 (24%)	49 (30%)	0.565
APOE ɛ4 carrier (N) (%)	9/39 (23%)	32/156 (21%)	0.826
ICH volume (ml)	49 (± 49)	16 (± 16)	<0.001*
MRI	(N = 38)	(N = 147)	
Lobar CMBs	9.0 (± 8.8)	3.3 (± 7.4)	<0.001*
Non-lobar CMBs	5.6 (± 9.1)	6.1 (± 8.4)	0.714
Total CMBs	14.6 (± 14.5)	9.4 (± 14.7)	0.051
Purely lobar CMBs (N) (%)	7 (18%)	4 (3%)	<0.001*
Mixed CMBs(N) (%)	22 (58%)	70 (48%)	0.521
Purely non-lobar CMBs (N) (%) (%)	4 (11%)	51 (35%)	0.004*
cSS (N) (%)	19 (50%)	6 (4%)	<0.001*
Lacune (N) (%)	20 (53%)	77 (52%)	0.978
WMH(N) (%)	21 (55%)	56 (38%)	0.056
PVS (N) (%)	26 (68%)	83 (56%)	0.182
brain atrophy(N) (%)	5 (13%)	10 (7%)	0.144
Post-stroke cognitive function			
MMSE (N = 134)	20.9 (± 8.2)	24.2 (± 6.8)	0.026*
MoCA (N = 119)	17.3 (± 8.3)	21.0 (± 6.8)	0.023*

ICH = intracerebral hemorrhage, N = Number, GCS = Glasgow Coma Scale, NIHSS = National Institutes of Health Stroke Scale. 1M = 1 month, CMBs = Cerebral microbleeds, cSS = cortical superficial siderosis, WMH = white matter hyperintensity, PVS = perivascular space, MMSE = Mini-Mental State Examination, MoCA = Montreal Cognitive Assessment. *Statistically significant.

**Fig 1:**
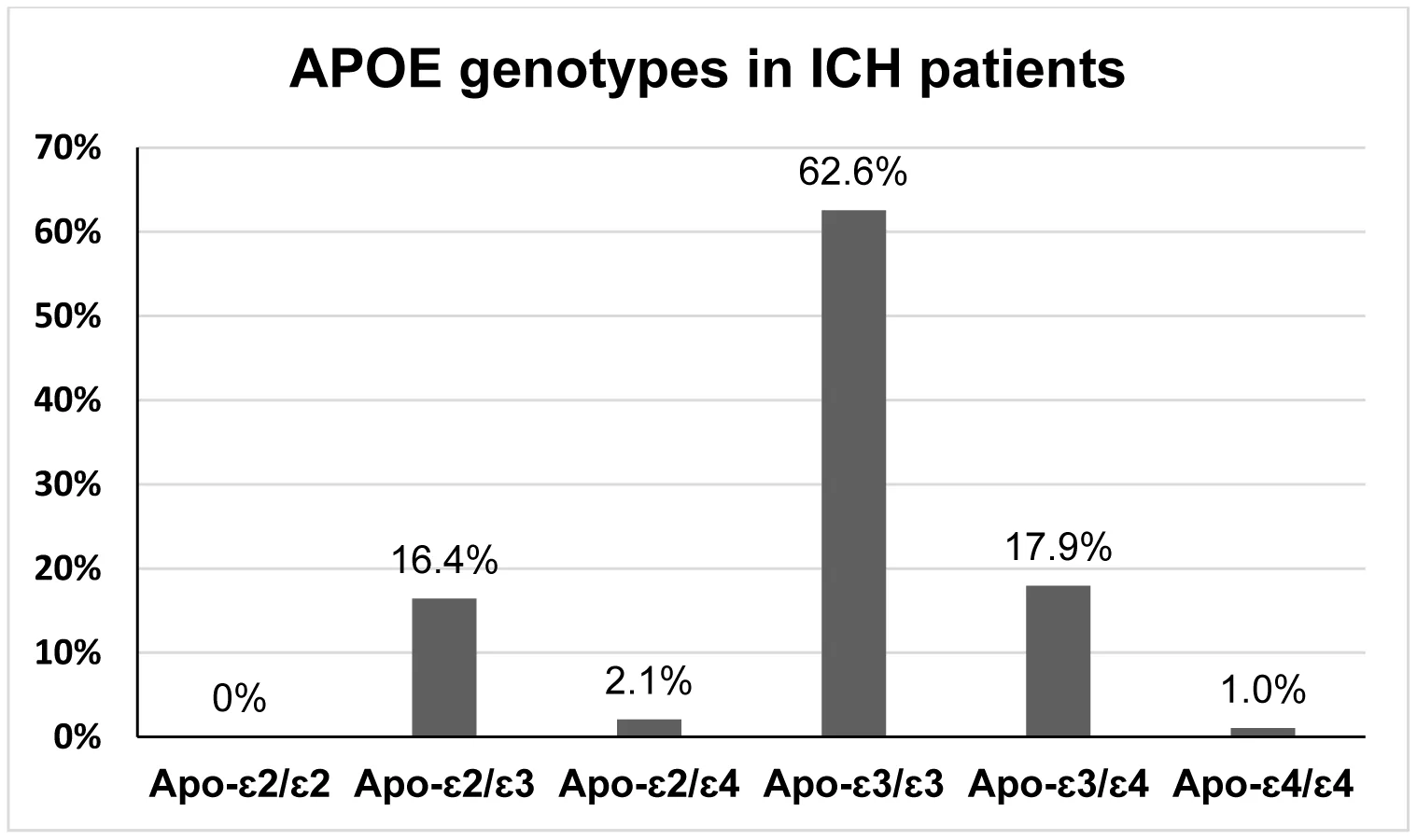
APOE genotypes in ICH patients. ICH = intracerebral hemorrhage.

For major risk factors, patients with non-lobar ICH had a significantly higher proportion of hypertension (98% vs 85%, p < 0.001), lower CAA (61% vs 0%, p < 0.001), and borderline of previous stroke than those with lobar ICH, but no significant differences in other vascular risk factors, pre-ICH antiplatelet or anticoagulant use (Table 1). The stronger association of hypertension with non-lobar ICH stayed significant after adjusting for age, sex and other risk factors (p = 0.007). To explore the genetic influence on ICH, 195 patients (97%) had available genetic results of APOE genotypes, and the most common APOE genotype was ɛ3/ɛ3 (63%) (Fig 1). The percentage of ɛ4 carrier (ɛ2/ɛ4, ɛ3/ɛ4, ɛ4/ɛ4) was 23% in lobar and 21% in non-lobar ICH patients, without significant difference in distribution of APOE genotypes (p = 0.826) or family history of stroke (p = 0.565).

In terms of etiologies of ICH, the most common cause in our patients was hypertension (76%), followed by CAA (14%), undetermined (4.5%), vascular structure lesion (4%) and medication (1.5%) [9]. When utilizing the Boston Criteria version 2.0, only 25 patients could be categorized into probable CAA group [10], accounting for 61% in lobar ICH. Comparing with non-CAA group, patients of CAA group had an average older age (mean age 72.1 vs 58.9 years, p < 0.001), a lower GCS (12.3 vs 14.0, p = 0.011), and a higher mean ICH score (1.2 vs 0.7, p = 0.030), while there was no significant difference in sex, mean NIHSS, major vascular risk factors, family history of stroke or APOE ɛ4 distribution (Table 2).

**Table 2:** Comparisons of clinical, genetic, neuroimage features, and post-stroke cognition between CAA and non-CAA ICH patients.

	CAA (N = 25)	Non-CAA (N = 177)	P
Mean age (year)	72.1 (±10.3)	58.9 (±11.8)	<0.001*
Sex (male) (N) (%)	13 (52%)	118 (67%)	0.181
GCS	12.3 (±3.9)	14.0 (±2.0)	0.040*
NIHSS	12.7 (±12.7)	8.4 (±7.0)	0.122
ICH score	1.2 (±1.2)	0.7 (**±** 0.9)	0.030*
1M case fatality	1 (4%)	2 (1%)	0.329
Hypertension(N) (%)	24 (96%)	169 (95%)	1.000
Diabetes (N) (%)	4 (16%)	44(25%)	0.330
Atrial fibrillation (N) (%)	3 (12%)	8 (5%)	0.141 242
Ischemic heart(N) (%)	1 (4%)	19 (11%)	0.478
Hypercholesterolemia(N) (%)	5 (20%)	51 (29%)	0.357
Hypertriglyceridemia (N) (%)	0 (0%)	13 (7%)	0.376
Smoking (N) (%)	12 (48%)	94 (53%)	0.632
Alcohol (N) (%)	7 (28%)	40 (23%)	0.550
Previous stroke(N) (%)	7 (28%)	24 (14%)	0.075
Pre-ICH antiplatelet(N) (%)	4 (16%)	20 (11%)	0.509
Pre-ICH anticoagulant(N) (%)	1 (4%)	5 (3%)	0.552
Family history of stroke (N) (%)	5 (20%)	53 (30%)	0.304
APOE ɛ4 carrier (N) (%)	7/24 (29%)	35/175 (20%)	0.332
Lobar ICH	25 (100%)	16 (9%)	<0.001*
ICH volume (ml)	60 (±56)	18 (±18)	<0.001*
MRI	**(N = 25)**	**(N = 160)**	
Lobar CMBs	9.9 (±9.5)	3.3 (± 7.2)	0.003*
Non-lobar CMBs	5.5 (±7.5)	6.1 (±8.7)	0.741
Total CMBs	15.4 (±12.6)	9.7 (±15.0)	0.074
Purely lobar CMBs (N) (%)	5 (20%)	6 (4%)	0.008*
Mixed CMBs(N) (%)	17 (68%)	92 (52%)	0.140
Purely non-lobar CMBs (N) (%)	2 (8%)	53 (33%)	0.010*
cSS (N) (%)	17 (68%)	8 (5%)	<0.001*
Lacune (N) (%)	14 (56%)	83 (52%)	0.701
WMH(N) (%)	14 (56%)	63 (39%)	0.117
PVS (N) (%)	18 (72%)	91 (57%)	0.153
brain atrophy(N) (%)	5 (20%)	10 (6%)	**0.035** ^*^
Post-stroke cognitive function			
MMSE (N = 134)	19.6(± 9.0)	24.1 (± 6.7)	0.016*
MoCA (N = 119)	15.8 (± 8.7)	20.9 (± 7.0)	0.017*

ICH = Intracerebral hemorrhage, N = Number, GCS = Glasgow Coma Scale, NIHSS = National Institutes of Health Stroke Scale. 1M = 1 month. CMBs = Cerebral microbleeds, cSS = cortical superficial siderosis, WMH = white matter hyperintensity, PVS = perivascular space, MMSE = Mini-Mental State Examination, MoCA = Montreal Cognitive Assessment. *Statistically significant.

### Neuroimaging features and early post-stroke cognition

Patients with lobar ICH usually had a larger ICH volume than those with non-lobar ICH (mean 49 ml vs 16 ml, p < 0.001) (Table 1). In patients with brain MRI, 85% of them had CMBs with various loading. Patients with lobar ICH usually had more lobar CMBs than non-lobar ones (mean 9.0 vs 3.3, p < 0.001), while there was no significant difference in non-lobar CMBs. Noticeably, more than half of lobar ICH had mixed CMBs over lobar and non-lobar areas (58%); only 18% had purely lobar CMBs. Regarding other neuroimaging markers in MRI, the lobar ICH group had a higher proportion of cSS (50% vs 4%, p < 0.001) than non-lobar group, but did not differ significantly in lacunes, WMH, PVS, and brain atrophy.

For early global cognitive performance after stroke, 134 ICH patients underwent global cognitive evaluation by MMSE around 1 month after ICH, and 119 patients had simultaneous MoCA assessment. Compared with non-lobar group, patients with lobar ICH generally had worse cognitive function in either MMSE (mean 20.9 vs 24.2, p = 0.026) or MoCA (mean 17.3 vs 21.0, p = 0.023) (Table 1). The proportion of cognitive impairment was also slightly higher in lobar ICH versus non-lobar ICH group by MMSE (41% vs 27%, p = 0.168) or MoCA (79% vs 49%, p = 0.011).

Similar findings were noted when comparing patients of CAA group with non-CAA groups according to Boston criteria version 2.0. ICH patients of CAA group generally had a higher proportion of lobar ICH (100% vs 9%, p < 0.001) and larger ICH volume (mean 60 vs 18, p < 0.001), more lobar CMBs (mean 9.9 vs 3.3, p = 0.003), cSS (68% vs 5%, p < 0.001) and brain atrophy (20% vs 6%, p = 0.035), along with worse post-stroke cognitive function in both MMSE (mean 19.6 vs 24.1, p = 0.016) and MoCA (mean 15.8 vs 20.9, p = 0.017) (Table 2). The proportion of cognitive impairment was also slightly higher in CAA versus non-CAA group by MMSE (41% vs 28%, p = 0.272) or MoCA (85% vs 52%, p = 0.036).

Additionally, we made comparisons between APOE ɛ4 and non-APOE ɛ4 carriers as APOE ɛ4 was reported to be a major genetic risk factor for ICH and dementia. Our results showed that post-stroke cognitive function was slightly worse in APOE ɛ4 than non-APOE ɛ4 carriers (MMSE 21.7 vs 24.1, p = 0.113; MoCA 17.4 vs 21.0, p = 0.037), while there was no significant difference in other clinical and neuroimaging profiles (S1 Table). The proportion of global cognitive impairment was also significantly higher in APOE ɛ4 than non-APOE ɛ4 carriers by MoCA (74% vs 50%, p = 0.039).

As 67 patients (33%) in this study could not undergo any cognitive assessment, we managed to compare these patients with those who were able to participate in cognitive evaluation (67%) (S2 Table). Patients who could not undergo cognitive assessment generally had a lower conscious level (GCS 13.0 vs 14.2, p = 0.001), more severe stroke (NIHSS 11.5 vs 7.7, p = 0.001) and ICH volume (29 ml vs 20 ml, p = 0.035), and a higher 1-month case fatality rate (4% vs 0%, p = 0.035) than other patients (S2 Table).

### Associated factors with early post-stroke cognition in ICH patients

We performed generalized linear regression models to clarify the possible associated factors with early global cognitive performance in ICH. In Model I for clinical factors, our analysis revealed age, education, NIHSS, GCS and ICH location were associated with early post-stroke cognitive function assessed by MMSE (Table 3). After adjustment for both clinical and image factors, Model II showed that age, education, NIHSS, GCS, and ICH volume were significantly associated factors, while total CMBs were borderline. Collinearity tests (VIF and tolerance) did not show strong correlations among these independent variables. Similar findings were noted when post-stroke cognition was assessed by MoCA in Model II (S3 Table). Sensitivity analysis (excluding 3 patients with diagnosis of dementia before ICH) disclosed consistent results.

**Table 3:** Models of clinical and neuroimage factors associated with post-stroke cognition in ICH patients (by MMSE).

	Model 1 (clinical factors)			Model 2 (clinical and neuroimaging factors)		
	B	95% CI	P value	B	95% CI	P value
**Age (year)**	−0.191	(−0.277, −0.105)	<0.001*	−0.174	(−0.255, −0.093)	<0.001*
**Education (year)**	0.387	(0.161, 0.613)	0.001*	0.279	(0.048, 0.510)	0.019*
**NIHSS**	−0.297	(−0.470, −0.124)	<0.001*	−0.228	(−0.392, −0.064)	0.007*
**GCS**	0.755	(0.166, 1.344)	0.013*	0.775	(0.199, 1.351)	0.009*
**ICH location**	3.996	(0.816, 7.177)	0.014*			
**CAA**	3.334	(−0.645, 7.313)	0.099			
**ICH volume**				−0.036	(−0.070 to −0.002)	0.040*
**MRI total CMBs**				−0.051	(−0.104 to 0.002)	0.066

ICH = Intracerebral hemorrhage, MMSE = Mini-Mental State Examination, Y = year, NIHSS = National Institutes of Health Stroke Scale, GCS = Glasgow Coma Scale, CMBs = Cerebral microbleeds, B = Beta coefficient, SE = Standard Error. *Statistically significant.

**Fig 2:**
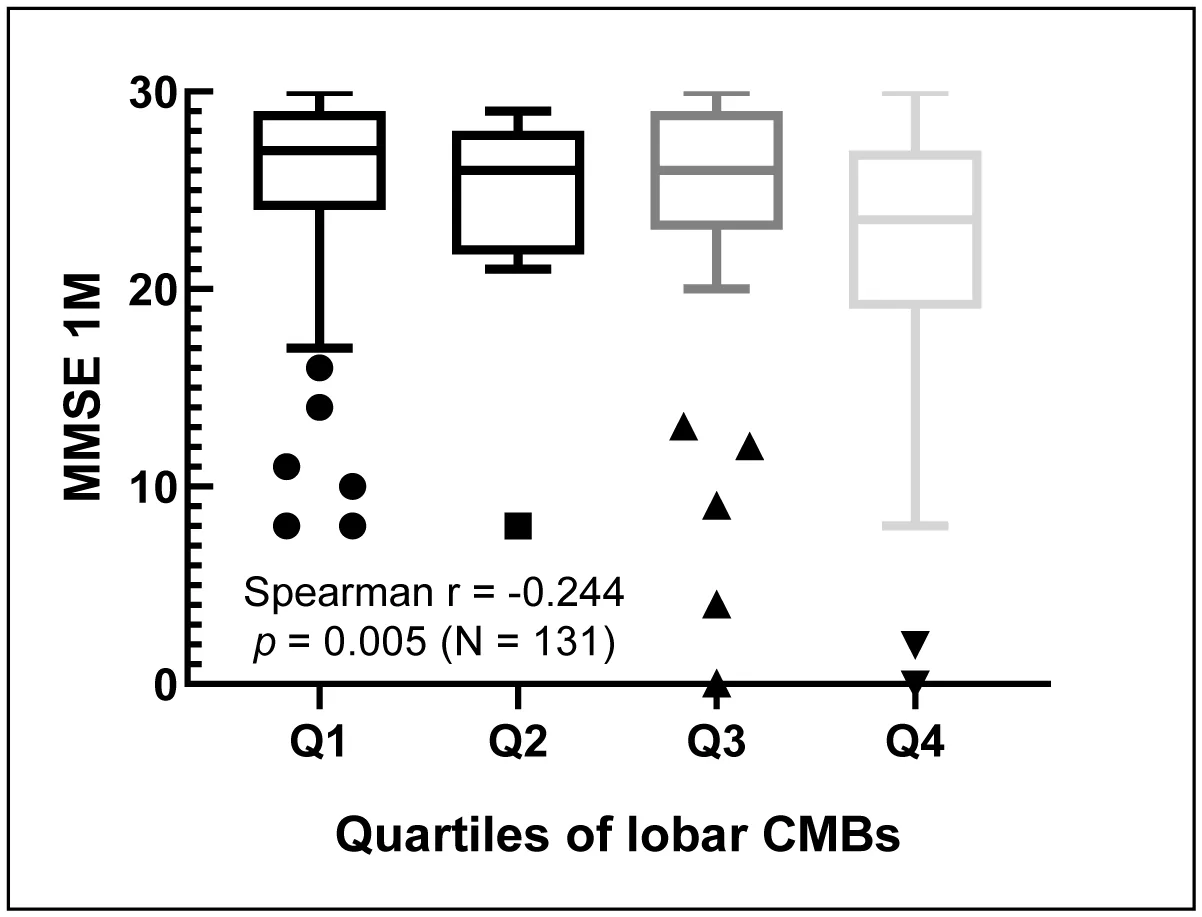
Associations of lobar CMBs with early post-stroke cognition in ICH patients (Box and Whisker Plots). Post-stroke cognition declined significantly with increasing lobar CMBs (measured by MMSE). CMBs = cerebral microbleeds, ICH = intracerebral hemorrhage, MMSE = Mini-Mental State Examination, M = month, N = number.

**Fig 3:**
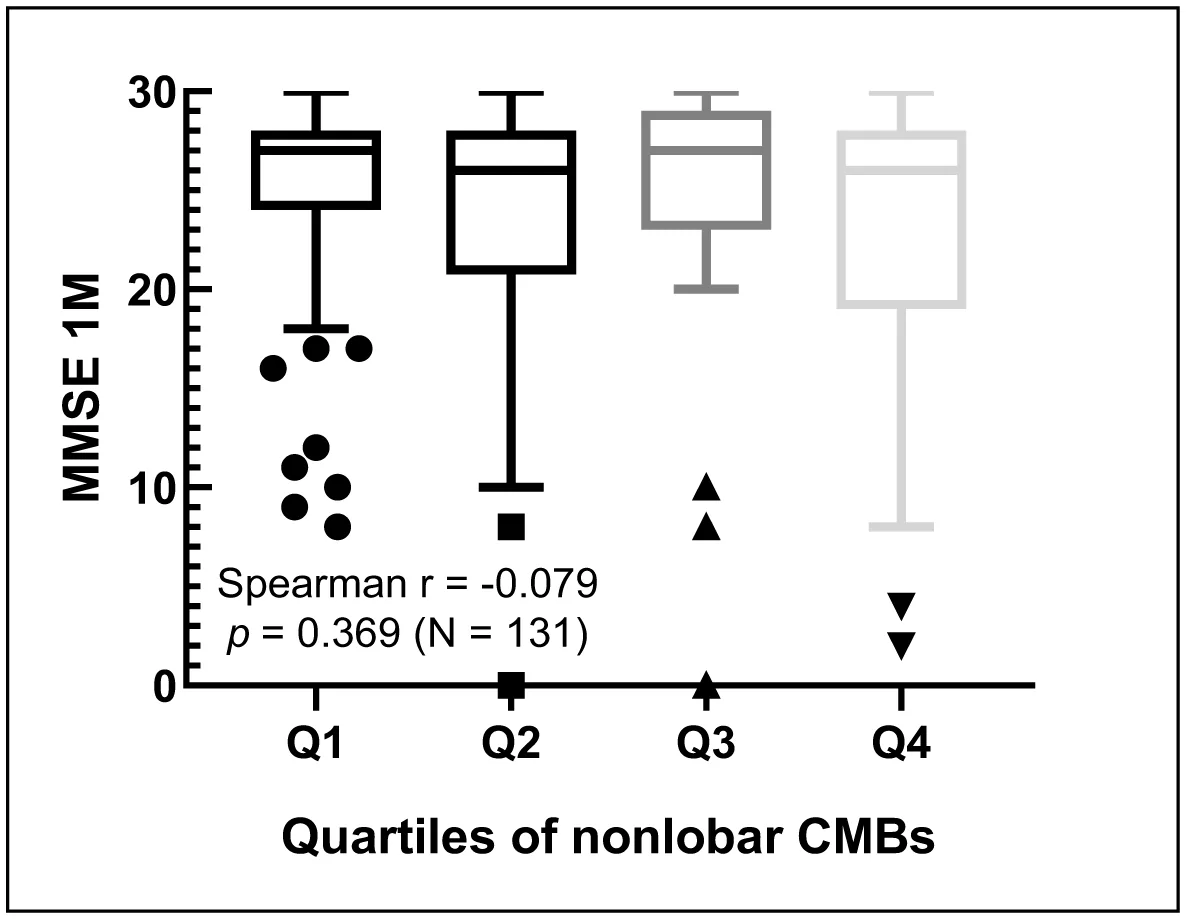
Associations of non-lobar CMBs with early post-stroke cognition in ICH patients (Box and Whisker Plots). Post-stroke cognition was not significantly associated with non-lobar CMBs (measured by MMSE). CMBs = cerebral microbleeds, ICH = intracerebral hemorrhage, MMSE = Mini-Mental State Examination, M = month, N = number.

To further explore the associations of CMB location and burden with post-stroke cognition, we conducted subgroup analysis for lobar and non-lobar CMBs. Box and Whisker Plots demonstrated that early global cognitive performance (assessed by MMSE) significantly declined with increasing lobar CMB loading (Fig 2, p = 0.005), but there was no significant association with non-lobar CMBs (Fig 3, p = 0.369). Post-stroke cognition measured by MoCA showed consistent results.

## Discussion

In our Chinese ICH patients, approximately two-thirds were men. The distribution of ICH was 80% in the non-lobar area and 20% in the lobar area. Patients with lobar ICH were generally older; they usually had a lower conscious level, a higher 1-month case fatality rate, a larger ICH volume, more lobar CMB burden and cSS, and worse early post-stroke cognitive function than non-lobar ICH patients, while non-lobar ICH patients had a higher proportion of hypertension than lobar patients, without significant difference in sex, NIHSS, distribution of other major risk factors or APOE genotypes. Similar findings were noted when comparing patients of CAA group with non-CAA groups according to Boston criteria version 2.0. CAA patients generally had an average older age, worse GCS, and a higher proportion of lobar ICH and larger ICH volume, more lobar CMBs, cortical superficial sclerosis and brain atrophy, along with worse post-stroke cognitive function than non-CAA patients. Additional analysis also revealed that APOE ɛ4 carriers had slightly worse cognitive function than non-APOE ɛ4 carriers. After adjusting for confounding factors, generalized linear regression models and subgroup analysis showed that early post-stroke cognition was associated with age, education, NIHSS, GCS, ICH volume, and lobar CMB loading.

The burden of stroke is heavy on Asian countries, including Chinese populations. It is the leading cause of death in China and the fourth in Taiwan [16,17]. In Western populations, spontaneous ICH accounts for around 10–12% of all strokes, but the proportion of ICH is nearly twofold in Chinese patients [3]. Although the incidence of ICH has decreased in urban China and Taiwan in recent years, it is still increasing in rural China [18–20]. The distribution of ICH seems different between populations as well. In our study, up to 80% of our Chinese ICH patients have deep ICH, higher than that in most Western studies [21,22]. It is probably related to the higher prevalence of hypertension and greater association with ICH in East Asians [23]. In our patients with lobar ICH, 61% fulfill the diagnosis of probable CAA and is the predominant mechanism. Lobar ICH patients have a slightly different clinical profile and a more severe early prognosis with a higher 1-month case fatality than deep ICH group, which are similar to the finding in another ICH study in Spain [24]. As for genetic risk factor, APOE e4 allele has been reported as an important risk factor for lobar ICH, associated with severe CAA vasculopathies [4,5]. However, there seem to be some racial differences. While APOEε4 is a major risk factor for lobar ICH in Western populations, it is less prevalent in Japanese, Africans and Hispanic people [25,26]. In our Chinese patients based in Taiwan, the proportion of ɛ4 carrier is relatively lower in lobar ICH as well, while the proportion of hypertension is higher than that in Western patients [21,22,26]. In addition, there is no significant difference in distribution of APOE genotypes between patients with lobar and non-lobar ICH. Further large-scale ICH studies, including more genetic analysis, are important.

CMBs in brain MRI are important markers for small vessel disease, implicating previous or recent cerebral hemorrhage, or increased risk of hemorrhage in the future [6–8]. They could help evaluate the possible underlying cerebral microangiopathy – strictly lobar CMBs or cSS tend to associate with CAA, while purely deep CMBs are more associated with hypertension angiopathy [6,27]. Interestingly, among our lobar ICH patients, only 18% have purely lobar CMBs, and more than half have mixed CMBs. When utilizing the Boston Criteria version 2.0 for CAA, just 61% of lobar ICH could fulfill the diagnosis of probable CAA [10]. Meanwhile, up to 85% of our patients with lobar ICH have hypertension. The high rates of hypertension and mixed CMBs in the lobar group suggest a significant role of hypertension and possible overlap of hypertensive angiopathy and CAA in our Chinese patients with lobar ICH.

As for cognitive impairment, it is commonly noted after ICH [28]. In our study, patients with lobar ICH or CAA group have worse early global cognitive performance measured by MMSE or MoCA than non-lobar ICH or non-CAA group, and APOE ɛ4 carriers present a slightly impaired cognitive function than non-APOE ɛ4 carriers. In addition, our subgroup analysis reveals that the early post-stroke cognition significantly declines with increasing lobar CMB loading, but not non-lobar CMBs. After adjustment for confounders, age, education, initial conscious level, stroke severity, ICH volume and lobar CMB loading are the associated factors for early post-stroke cognition. In literature, the relationship between CMBs and cognition is conflicting. Some studies show that CMBs are associated with cognitive impairment and dementia, while others do not reveal increasing risk of dementia [29,30]. Regarding APOE genotypes, APOE ɛ4 is an important risk factor for Alzheimer disease, which is also reported to associate with dementia before and after stroke [31]. Further cognitive follow-up could help us clarify the impacts of genetics and CMBs on long-term post-stroke cognitive outcome.

Our study has several strengths. First, we prospectively recruited acute spontaneous ICH patients, acquiring important clinical information and doing APOE genetic study, without selection of age, sex or socioeconomic status. Second, recruitment of acute ICH patients was based on a standard definition of stroke and ICH on brain CT in all. Over 90% of them had additional brain MRI/MRA including SWI to assess CMBs and other novel neuroimaging markers. Third, we made comparisons between different ICH locations, etiologies and APOE genotypes, used multivariable generalized linear regression models to investigate the association of clinical and image variables with early post-stroke cognition, and conducted sensitivity analysis to exclude patients with pre-existing dementia. Furthermore, we carried out subgroup analysis to examine the association of lobar and non-lobar CMBs with early post-stroke cognition. There were some limitations. This was a prospective, hospital-based study in Taiwan without a large sample size, which might not be fully representative of all Chinese or Asian ICH patients. In particular, the small sample size of probable CAA group precluded us from other subgroup analysis as it might reduce the statistical power. Also, not all patients could have brain MRI or cognitive assessment after ICH as a couple of them had unstable hemodynamics, poor conscious level, or early mortality after ICH. Since patients who could not participate in the cognitive assessment usually had a more severe stroke, this could be a systematic selection bias and potentially underestimate frequency of cognitive impairment in patients after ICH. In addition, cognitive assessment in this study was performed by MMSE and MoCA and these two tests could not provide comprehensive cognitive assessment, especially for some specific cognitive domains. Finally, cognitive evaluation was performed between the first and second month after ICH, which could not represent long-term cognitive outcome. Further clinical and cognitive follow-up are needed.

## Conclusion

ICH is a multifactorial disease influenced by various vascular and genetic risk factors, pathologies, and precipitating factors across populations [32]. Herein we report the characteristics and differences in clinical and imaging features, major vascular and genetic risk factors, and early global cognitive performance between different ICH locations and etiologies in Chinese patients, who have a higher incidence rate of ICH in the world. Also, we investigated clinical and neuroimaging factors associated with early global cognitive function after stroke. Further large-scale ICH studies, including more genetic analyses, are important. In addition, risk factors associated with early or delayed cognitive impairment after ICH may be different. Thus, further clinical and cognitive follow-up could help us clarify the impact of these associated factors on long-term cognitive and functional outcomes in ICH patients.

## Supporting information

S1 Table Comparisons between APOEɛ4 and non-APOEɛ4 carriers in ICH patients. (DOCX)

S2 Table Characteristics of ICH patients with and without cognitive assessment. (DOCX)

S3 Table Models of clinical and image factors associated with post-stroke cognition in ICH patients (by MoCA). (DOCX)

S1 Fig Study flow chart of inclusion of acute spontaneous intracerebral hemorrhage (ICH) patients. (DOCX)
