# Supplementary material for: Clinical and neuroimaging features, and associations with early post-stroke cognition in patients with intracerebral hemorrhage in Taiwan
Source: PLoS One. 2026 Sep 21;21(9):e0358545. doi: 10.1371/journal.pone.0358545 (PMC13592605; doi:10.1371/journal.pone.0358545)
Supplement: Supplementary file 1 [file pone.0358545.s001.docx]

**S1 Table. Comparisons between APOEɛ4 and non-APOEɛ4 carriers in ICH patients.**

|  |  | APOEɛ4  (N=42, 22%) | Non-APOEɛ4  (N=153, 78%) | P |
| --- | --- | --- | --- | --- |
|  | Mean age (year) | 59.7(±14.2) | 60.9 (±12.0) | 0.574 |
|  | Sex (male) (%) | 23 (55%) | 103 (67%) | 0.132 |
|  | GCS | 13.5 (±2.8) | 13.9 (± 2.2) | 0.369 |
|  | NIHSS | 11.1 (± 9.6) | 8.1 (±7.2) | 0.067 |
|  | ICH score | 0.9 (**±**0.9) | 0.7 (**±** 0.9) | 0.204 |
|  | 1m case fatality | 0 (0%) | 2 (1.3%) | 0.623 |
|  | lobar ICH | 9 (21%) | 30 (20%) | 0.794 |
|  | Non-lobar ICH | 33 (79%) | 123 (80%) | 0.794 |
| Hypertension(N) (%) | | 42 (100%) | 145 (95%) | 0.205 |
| Diabetes (N) (%) | | 9 (21%) | 37 (24%) | 0.710 |
| Atrial fibrillation (N) (%) | | 3 (7%) | 8 (5%) | 0.706  242 |
| Ischemic heart(N) (%) | | 3 (7%) | 15 (10%) | 0.768 |
| Hypercholesterolemia(N) (%) | | 12 (29%) | 42 (27%) | 0.886 |
| Hypertriglycemia (N) (%) | | 4 (10%) | 9 (6%) | 0.483 |
| Smoking (N) (%) | | 24 (57%) | 78 (51%) | 0.326 |
| Alcohol (N) (%) | | 9 (21%) | 33 (22%) | 0.913 |
| Previous stroke(N) (%) | | 7 (17%) | 23 (15%) | 0.795 |
| Pre-ICH antiplatelet(N) (%) | | 3 (7%) | 20 (13%) | 0.420 |
| Pre-ICH anticoagulation(N) (%) | | 3 (7%) | 3 (2%) | 0.115 |
| Family history of stroke (N) (%) | | 14 (33%) | 42 (28%) | 0.456 |
| ICH volume (ml) | | 25 (±28) | 22 (±30) | 0.554 |
|  | MRI | **(N=38)** | **(N=142)** |  |
|  | Lobar CMBs | 4.8 (±7.9) | 4.4 (± 8.8) | 0.817 |
|  | Non-lobar CMBs | 6 (±7.6) | 6 (±8.9) | 0.986 |
|  | Total CMBs | 10.8 (±12.7) | 10.4 (±15.6) | 0.886 |
|  | Purely lobar CMBs (N) (%) | 2 (5%) | 7 (5%) | 0.377 |
|  | mixed cmbs(N) (%) | 22 (58%) | 67 (47%) | 0.241 |
|  | Purely non-lobar CMBs (N) ( (%) (%) | 10 (26%) | 42 (30%) | 0.694 |
|  | cSS (N) (%) | 7 (18%) | 17 (12%) | 0.299 |
|  | Lacune (N) (%) | 17 (45%) | 76 (54%) | 0.336 |
|  | WMH(N) (%) | 13 (34%) | 62 (44%) | 0.294 |
|  | PVS (N) (%) | 22 (58%) | 84 (59%) | 0.889 |
|  | brain atrophy(N) (%) | 2 (5%) | 13 (9%) | 0.741 |
|  | Post-stroke cognitive function |  |  |  |
|  | MMSe (N=131) | 21.7(± 8.3) | 24.1 (± 6.6) | 0.113 |
|  | MoCA (N=117) | 17.4 (± 8.5) | 21.0 (± 6.9) | **0.037*** |

ICH=Intracerebral hemorrhage, N=Number, GCS=Glasgow Coma Scale, NHISS=National Institutes of Health Stroke Scale. 1M=1 month. CMBs=Cerebral microbleeds, cSS=cortical superficial sclerosis, WMH=white matter hyperintensity, PVS=perivascular space, Mini-Mental Status Examination, MoCA= Montreal Cognitive Assessment. *Statistically significant.
