# Supplementary material for: Clinical and neuroimaging features, and associations with early post-stroke cognition in patients with intracerebral hemorrhage in Taiwan
Source: PLoS One. 2026 Sep 21;21(9):e0358545. doi: 10.1371/journal.pone.0358545 (PMC13592605; doi:10.1371/journal.pone.0358545)
Supplement: Supplementary file 2 [file pone.0358545.s002.docx]

**S2 Table. Clinical characteristics of ICH patients with and without cognitive assessment.**

|  |  | With cognitive assessment  (N=135, 67%) | Without cognitive assessment  (N=67, 33%) | P |
| --- | --- | --- | --- | --- |
|  | Mean age (year) | 59.5(±11.2) | 62.6 (±14.4) | 0.130 |
|  | Sex (male) (%) | 83 (61%) | 48 (72%) | 0.154 |
|  | GCS | 14.2 (±2.1) | 13.0 (±2.7) | **0.001*** |
|  | NIHSS | 7.7 (± 7.3) | 11.5 (±8.7) | **0.001*** |
|  | ICH score | 0.6 (**±**0.8) | 1.1 (**±** 1.1) | **<0.001*** |
|  | 1m case fatality | 0 (0%) | 3 (4%) | **0.035*** |
|  | lobar ICH | 29 (21%) | 12 (18%) | 0.552 |
|  | Non-lobar ICH | 106 (79%) | 55 (82%) | 0.552 |
| Hypertension(N) (%) | | 129 (96%) | 64 (96%) | 1.000 |
| Diabetes (N) (%) | | 27 (20%) | 21 (31%) | 0.075 |
| Atrial fibrillation (N) (%) | | 9 (7%) | 2 (3%) | 0.344 |
| Ischemic heart(N) (%) | | 11 (8%) | 9 (13%) | 0.236 |
| Hypercholesterolemia(N) (%) | | 44 (33%) | 12 (18%) | **0.028*** |
| Hypertriglycemia (N) (%) | | 10 (7%) | 3 (4%) | 0.551 |
| Smoking (N) (%) | | 69 (51%) | 37 (55%) | 0.582 |
| Alcohol (N) (%) | | 29 (21%) | 18 (27%) | 0.394 |
| Previous stroke(N) (%) | | 20 (15%) | 11 (16%) | 0.766 |
| Pre-ICH antiplatelet(N) (%) | | 16 (12%) | 8 (12%) | 0.985 |
| Pre-ICH anticoagulation(N) (%) | | 5 (4%) | 1 (1%) | 0.666 |
| Family history of stroke (N) (%) | | 48 (36%) | 10 (15%) | **0.002*** |
| ICH volume (ml) | | 20 (±27) | 29 (±33) | **0.035*** |

ICH=Intracerebral hemorrhage, N=Number, GCS=Glasgow Coma Scale, NHISS=National Institutes of Health Stroke Scale. 1M=1 month. *Statistically significant.
