# Supplementary material for: Clinical and neuroimaging features, and associations with early post-stroke cognition in patients with intracerebral hemorrhage in Taiwan
Source: PLoS One. 2026 Sep 21;21(9):e0358545. doi: 10.1371/journal.pone.0358545 (PMC13592605; doi:10.1371/journal.pone.0358545)
Supplement: Supplementary file 3 [file pone.0358545.s003.docx]

**S3 Table. Models of clinical and image factors associated with post-stroke cognition in ICH patients (by MoCA).**

|  | **Model 1**  **(clinical factors)** | | | | **Model 2**  **(clinical and image factors)** | | | | |
| --- | --- | --- | --- | --- | --- | --- | --- | --- | --- |
|  | **B** | **SE** | **95% CI** | **P value** | | **B** | **SE** | **95% CI** | **P value** |
| **Age (year)** | -0.259 | 0.048 | (-0.354, -0.164) | <0.001* | | -0.233 | 0.048 | (-0.328, -0.138) | <0.001* |
| **Education (year)** | 0.471 | 0.131 | (0.211, 0.731) | 0.001* | | 0.481 | 0.136 | (0.211, 0.751) | 0.001* |
| **GCS** | 1.559 | 0.260 | (1.043, 2.075) | <0.001* | | 1.595 | 0.257 | (1.085, 2.105) | <0.001* |
| **HTG** | -3.833 | 1.805 | (-7.413, -0.253) | 0.036* | | -3.590 | 1.815 | (-7.194, 0.014) | 0.051 |
| **MRI CMBs total** |  |  |  |  | | -0.098 | 0.037 | (-0.171, -0.025) | 0.009* |

ICH= Intracerebral hemorrhage, NPT= Neuropsychological tests, GCS=Glasgow Coma Scale, HTG= Hypertriglycemia, CMBs= Cerebral microbleeds, B=Beta coefficient, SE=Standard Error, CI=Confidence Interval. *Statistically significant.
