# Supplementary material for: Clinical and neuroimaging features, and associations with early post-stroke cognition in patients with intracerebral hemorrhage in Taiwan
Source: PLoS One. 2026 Sep 21;21(9):e0358545. doi: 10.1371/journal.pone.0358545 (PMC13592605; doi:10.1371/journal.pone.0358545)
Supplement: Supplementary file 4 [file pone.0358545.s004.docx]

**S1 Fig. Study flow chart of inclusion of acute spontaneous intracerebral hemorrhage (ICH) patients.**

Collect clinical information and arrange genetic study (N=202)

Excluding traumatic ICH, pure subarachnoid/subdural/epidural/intraventricular hemorrhage, post-infarct hemorrhagic transformation, tumor, non-cerebrovascular causes, or no available informed consent

(N=53)

Arrange brain MRI/SWI/MRA

(N=185)

Acute ICH patients within 1 month recruited from Cardinal Tien and National Taiwan Hospitals

(N=255)

Not suitable for brain MRI due to unstable hemodynamics (N=17)

(N=185)

Arrange neuropsychological tests 1 month after ICH

(N=134)

Not suitable for neuropsychological tests due to early death or uncooperative status (N=68)
