# Supplementary material for: Clinical and neuroimaging features, and associations with early post-stroke cognition in patients with intracerebral hemorrhage in Taiwan
Source: PLoS One. 2026 Sep 21;21(9):e0358545. doi: 10.1371/journal.pone.0358545 (PMC13592605; doi:10.1371/journal.pone.0358545)
Supplement: Supplementary file 5 [file pone.0358545.s006.docx]

2026 PLOS ONE – Responses to Reviewers’ comments on

***"* *Clinical and neuroimaging features, and*** ***associations with early post-stroke cognition in patients with intracerebral hemorrhage in Taiwan***

***"*** *[PONE-D-26-33405] - [EMID:ded16d32aa5e4d58]*

Dear Academic Editor Jinglu Ai and Reviewers,

Many thanks for your positive feedback and for giving us the opportunity to revise our manuscript. We had made corresponding changes based on PLOS ONE's style requirements, Editor and reviewer’s suggestions, and prepared a letter “Response to Reviewers” below, replying to Reviewers’ comments point by point and stating the page and paragraph number of specific changes in the revised manuscript. Also, we have updated our Funding Statement and Data Availability Statement in the cover letter.

**Journal Requirements:**
1. Please ensure that your manuscript meets PLOS ONE's style requirements, including those for file naming. The PLOS ONE style templates can be found at 
<https://journals.plos.org/plosone/s/file?id=wjVg/PLOSOne_formatting_sample_main_body.pdf> and 
<https://journals.plos.org/plosone/s/file?id=ba62/PLOSOne_formatting_sample_title_authors_affiliations.pdf>

**Author’s reply:**

Thanks for your reminder. We have checked journal formatting guidelines to meet PLOS ONE’s style.

2. Thank you for stating the following in the Acknowledgments Section of your manuscript: 
“This work was partly supported by the Cardinal Tien Hospital Research Grants (CTH 113A-2213, CTH 114A-2212; C.-F. Tsai). The funder had no role in the study design, data collection and analysis, decision to publish, or preparation of the manuscript”
We note that you have provided funding information that is not currently declared in your Funding Statement. However, funding information should not appear in the Acknowledgments section or other areas of your manuscript. We will only publish funding information present in the Funding Statement section of the online submission form. 
Please remove any funding-related text from the manuscript and let us know how you would like to update your Funding Statement. Currently, your Funding Statement reads as follows: 
“This work was partly supported by the Cardinal Tien Hospital Research Grants (CTH 113A-2213, CTH 114A-2212; C.-F. Tsai). The funder had no role in the study design, data collection and analysis, decision to publish, or preparation of the manuscript.”

Please include your amended statements within your cover letter; we will change the online submission form on your behalf.

**Author’s reply:**

We have removed any funding-related text from the manuscript, updated our Funding Statement below, and included the amended statement in our cover letter.

3. Thank you for stating in your Funding Statement: 
“This work was partly supported by the Cardinal Tien Hospital Research Grants (CTH 113A-2213, CTH 114A-2212; C.-F. Tsai). The funder had no role in the study design, data collection and analysis, decision to publish, or preparation of the manuscript.”

Please provide an amended statement that declares *all* the funding or sources of support (whether external or internal to your organization) received during this study, as detailed online in our guide for authors at <http://journals.plos.org/plosone/s/submit-now>.  Please also include the statement “There was no additional external funding received for this study.” in your updated Funding Statement. 
Please include your amended Funding Statement within your cover letter. We will change the online submission form on your behalf.

**Author’s reply:**

Thanks for your suggestion. We have updated the Funding Statement: “This work was partly supported by the Cardinal Tien Hospital Research Grants (CTH 113A-2213, CTH 114A-2212; C.-F. Tsai). There was no additional external funding received for this study. All the funding or sources of support received during this study had no role in study design, data collection and analysis, decision to publish, or preparation of the manuscript.” Also, we have included the amended Funding Statement in our cover letter.

4. Thank you for stating the following financial disclosure:

“This work was partly supported by the Cardinal Tien Hospital Research Grants (CTH 113A-2213, CTH 114A-2212; C.-F. Tsai). The funder had no role in the study design, data collection and analysis, decision to publish, or preparation of the manuscript.”
We note that one or more of the authors is affiliated with the funding organization, indicating the funder may have had some role in the design, data collection, analysis or preparation of your manuscript for publication; in other words, the funder played an indirect role through the participation of the co-authors. If the funding organization did not play a role in the study design, data collection and analysis, decision to publish, or preparation of the manuscript and only provided financial support in the form of authors' salaries and/or research materials, please do the following:

1. Review your statements relating to the author contributions, and ensure you have specifically and accurately indicated the role(s) that these authors had in your study. These amendments should be made in the online form.

2. Confirm in your cover letter that you agree with the following statement, and we will change the online submission form on your behalf:

“The funder provided support in the form of salaries for authors [insert relevant initials], but did not have any additional role in the study design, data collection and analysis, decision to publish, or preparation of the manuscript. The specific roles of these authors are articulated in the ‘author contributions’ section.

**Author’s reply:**

Yes, we have reviewed the statement related to the author contributions and have confirmed in our cover letter that we agree with the following statement: “The funder provided support in the form of salaries for authors [CT, YW, and PY], but did not have any additional role in the study design, data collection and analysis, decision to publish, or preparation of the manuscript. The specific roles of these authors are articulated in the ‘author contributions’ section".

5. In the online submission form you indicate that your data is not available for proprietary reasons and have provided a contact point for accessing this data. Please note that your current contact point is a co-author on this manuscript. According to our Data Policy, the contact point must not be an author on the manuscript and must be an institutional contact, ideally not an individual. Please revise your data statement to a non-author institutional point of contact, such as a data access or ethics committee, and send this to us via return email. Please also include contact information for the third party organization, and please include the full citation of where the data can be found.

**Author’s reply:**

We have revised our Data Availability Statement in the cover letter as follows - Data generated or analyzed during this study are included in the manuscript and its Supporting Information files. Because of ethical and privacy consideration, further enquiries for other relevant data can be made to Institution Review Board of the Cardinal Tien Hospital (<https://www.cth.org.tw/?aid=52&pid=70>) (irb.cth@gmail.com) for researchers who meet the criteria for access to confidential data.

6. Your ethics statement should only appear in the Methods section of your manuscript. If your ethics statement is written in any section besides the Methods, please move it to the Methods section and delete it from any other section. Please ensure that your ethics statement is included in your manuscript, as the ethics statement entered into the online submission form will not be published alongside your manuscript.

**Authors’ reply:**

Thank you. We have placed ethic statement in the Methods section and removed it from any other section (first paragraph, page 6).

7. If the reviewer comments include a recommendation to cite specific previously published works, please review and evaluate these publications to determine whether they are relevant and should be cited. There is no requirement to cite these works unless the editor has indicated otherwise.

**Author’s reply:**

Thanks for your reminder. We have reviewed and evaluated these publications carefully and only cited the paper which is relevant to this study (Reference 24).

8. Please review your reference list to ensure that it is complete and correct. If you have cited papers that have been retracted, please include the rationale for doing so in the manuscript text, or remove these references and replace them with relevant current references. Any changes to the reference list should be mentioned in the rebuttal letter that accompanies your revised manuscript. If you need to cite a retracted article, indicate the article’s retracted status in the References list and also include a citation and full reference for the retraction notice.

**Author’s reply:**

We have reviewed our references, marked the added or changed references in the “Response to Reviewers” and our revised manuscript. Thank you.

**Reviewers’ comments**

**Reviewer #1:** The authors present the results of a prospective, hospital-based study from two large hospitals in Taiwan aimed to make comparisons between different intracerebral hemorrhage (ICH) locations and etiologies, and to investigate the factors associated with early poststroke cognition. A total of 202 acute spontaneous ICH patients were included in the study. The authors demonstrated differences in clinical and imaging features, major risk factors, and early post-stroke cognitive function between different locations and etiologies. Age, education, stroke severity, ICH volume and lobar CMB burden were the associated factors for early post-stroke cognition. The study is potentially interesting, but can be improved if the following considerations are addressed:

1.The text should place greater emphasis on the fact that acute lobar cerebral hemorrhages present a different clinical profile and a more severe early prognosis than deep subcortical intracerebral hemorrhages and should add that non-hypertensive mechanisms of intracerebral hemorrhages predominate in the lobar location. Please compare their findings with data from a study reported in Biomedicines 2023 Jan 16;11(1):223.

**Author’s reply:**

Many thanks for your valuable suggestions. We have revised our manuscript to put greater emphasis on the fact that patients with lobar cerebral hemorrhages have a different clinical profile and a more severe early prognosis with a higher 1-month case-fatality than those with deep intracerebral hemorrhages (first paragraph, page 18). Also, we made comparisons with findings in another ICH study mentioned above and added it in our references (Biomedicines 2023 Jan 16;11(1):223, Reference 24). In addition, we have added the statement that 61% of patients with lobar ICH fulfill the diagnosis of probable CAA and is the predominate mechanism (first paragraph, page 18).

2.The authors have certainly and appropriately mentioned in the text the clinical severity of intracerebral hemorrhages. However, it would be interesting to mention also that there is a subgroup of hypertensive cerebral hemorrhages of small size of the lesion, which clinically manifest as a lacunar syndrome (“hemorrhagic pure motor stroke”). This subgroup accounts for 3.3% of cerebral hemorrhages and are associated with a more favorable prognosis as compared to the remaining group of intracerebral hemorrhages, usually without in-hospital deaths and symptom-free at discharge in 25% of cases, as described in a clinical series of this condition (see and include this supporting reference: Eur J Neurol. 2007 Feb;14(2):219-23. doi: 10.1111/j.1468-1331.2006.01660.x. PMID: 17250733.). Did the authors consider this in their study?

**Author’s reply:**

Thanks for your question. That’s indeed interesting. However, after reading the paper published on 2007 Eur J Neurol and checking the records of our study, we did not find “hemorrhagic pure motor stroke” in our patients. It’s probably a very infrequent stroke subtype and our sample size is limited. We are glad to pay more attention to this point in the future.

3.It would be interesting to add the results in the subgroup of very old patients (aged 85 years or older) given that demographics, risk factors and prognosis are quite different in this age group of stroke patients (Evidence supporting this point should be included after searching in PubMed for suitable publications). Did the authors consider this in their study?

**Author’s reply:**

Thanks for your suggestion. We have managed to make a table to compare the very old patients (aged 85 years or older) versus others below. The very old patients with ICH have a significantly higher proportion of atrial fibrillation than others, while the distribution of sex, GCS, NIHSS, ICH volume, other vascular risk factors, and 1-month case fatality do not differ significantly between these two groups. However, there are only 4 very old patients (2%) in our study, which may reduce statistical power. The results should be interpreted with caution.

**Table. Comparisons between the very old patients and other patients with ICH**

|  |  | **<85 years**  **(N=198, 98%)** | **≥85 years**  **(N=4, 2%)** | **P** |
| --- | --- | --- | --- | --- |
|  | **Mean age (year)** | 60.0(±11.8) | 89.5 (±7.7) | **<0.001*** |
|  | **Sex (male) (%)** | 130 (66%) | 1 (25%) | 0.126 |
|  | **GCS** | 13.8 (±2.4) | 13.3 (± 2.2) | 0.152 |
|  | **NIHSS** | 8.8 (± 7.9) | 17.3 (±11.1) | 0.107 |
|  | **ICH score** | 0.7 (**±**0.9) | 1.5 (**±** 1.0) | 0.069 |
|  | **1m case fatality** | 3 (2%) | 0 (0%) | 1.000 |
| **Hypertension(N) (%)** | | 189 (96%) | 4 (100%) | 1.000 |
| **Diabetes (N) (%)** | | 47 (24%) | 1 (25%) | 1.000 |
| **Atrial fibrillation (N) (%)** | | 9 (5%) | 2 (50%) | **0.015*** |
| **Ischemic heart(N) (%)** | | 20 (10%) | 0 (0%) | 1.000 |
| **Hypercholesterolemia(N) (%)** | | 56 (28%) | 0 (0%) | 0.577 |
| **Hypertriglycemia (N) (%)** | | 13 (7%) | 0 (0%) | 1.000 |
| **Smoking (N) (%)** | | 105 (53%) | 1 (25%) | 0.348 |
| **Alcohol (N) (%)** | | 46 (23%) | 1 (25%) | 1.000 |
| **Previous stroke(N) (%)** | | 30 (15%) | 1 (25%) | 0.489 |
| **Pre-ICH antiplatelet(N) (%)** | | 24 (12%) | 0 (0%) | 1.000 |
| **Pre-ICH anticoagulation(N) (%)** | | 5 (3%) | 1 (25%) | 0.114 |
| **Family history of stroke (N) (%)** | | 57 (29%) | 1 (25%) | 1.000 |
| **ICH volume (ml)** | | 23 (±29) | 31 (±30) | 0.567 |

4.A brief concluding comment on other possible lines of future research on the presented topic would be appreciated.

**Author’s reply:**

We are grateful for your comments. We have added the possible directions of further research in the Conclusion Section (first paragraph, page 21).

**Reviewer #2**: This study investigated the clinical characteristics, risk factors, radiological findings, and early cognitive performance of patients diagnosed with intracerebral hemorrhage in Taiwan, according to the localization of the hemorrhage. It is a prospective, double-center observational study. The strengths of the study are its prospective nature, the inclusion of genetic analyses, and the combined evaluation of neuroimaging findings. Overall, the study design, the tests used, and the statistical methods applied are appropriate. The data are consistent with the results obtained. However, I believe that the methodology needs to be made more transparent and the interpretation of some results needs to be strengthened. I would like to state that fulfilling the following suggestions will increase the scientific validity of this study and will be beneficial for readers to better understand it.

1. A total of 202 patients were included in the study, but it was found that MMSE and MoCA tests could only be performed on 134 patients and 119 patients, respectively. The authors stated that part of the reason for this was hemodynamic instability, impaired consciousness, and early mortality. However, the clinical characteristics (age, NIHSS, GCS, ICH volume, mortality, etc.) of patients who could not be included in the cognitive assessment were not specified, and they were not compared with those of other patients. It is highly probable that patients who could not participate in the cognitive assessment had a more severe stroke. Therefore, a systematic selection bias may have occurred in the cognitive analyses, and the results obtained may underestimate the actual frequency of cognitive impairment. The authors could briefly present the characteristics of patients who could not undergo cognitive assessment, or at least discuss the possible impact of this on the results in more detail in the Discussion section. I believe this is important to strengthen the study.

**Author’s reply:**

Many thanks for your intelligent insight and suggestion. We agree with your opinion that patients who could not participate in the cognitive assessment might have a more severe stroke. We have added a new table to summarize the important characteristics of the patient who could not undergo cognitive evaluation (33%) and compared with those with cognitive assessment (67%) (S2 Table) and briefly described findings in the Results section (paragraph 2, page 15). Also, we have stated the possible systematic selection bias and the potentially underestimated frequency of cognitive impairment in this study in the Discussion section (paragraph 1, page 20).

2. The phrase "post-stroke cognition" is frequently used in many parts of the article, including the title and aims. However, cognitive assessment was performed only with MMSE and MoCA. While these two tests are widely used, valid, and reliable tools for global cognitive screening, they cannot comprehensively assess executive functions, attention, processing speed, working memory, and other specific cognitive domains that are frequently affected, especially after intracerebral hemorrhage. Therefore, these tests alone do not provide comprehensive information regarding cognitive assessment. For this reason, it would be more appropriate to interpret the results in the context of early global cognitive performance measured by MMSE and MoCA, rather than "post-stroke cognition." It is recommended that this limitation be emphasized more clearly in the Discussion and Conclusion sections.

**Author’s reply:**

Thanks for your comments. We fully agree with your opinion that cognitive assessment in this study was performed by MMSE and MoCA and these two tests could not provide comprehensive cognitive assessment, especially for some specific cognitive domains. We have revised the text and added this limitation in the Discussion (first paragraph, page 20) and Conclusion sections (second paragraph, page 20).

3. The authors state that they used a generalized linear regression model for multivariate analysis. In general, this appears to be a suitable method. However, if the approach by which the variables were included in the model (clinical significance, literature information, or univariate analysis results) were explained more clearly, and the basic characteristics of the model were briefly stated, the methodology and analysis of the study would be stronger and more transparent.

**Author’s reply:**

We thank you for this point and are glad to explain more clearly in the Methods Section. To investigate the associations with early global cognitive function in ICH patients, we included clinical and neuroimaging variables of clinical significance and literature information into generalized liner regression models for multivariate analysis and used stepwise selection method in the Method section (second paragraph, page 8; first paragraph, page 9).

4. Some variables included in the multivariate model (e.g., GCS, NIHSS, and ICH volume) may be considered clinically related to each other. If evaluated, it should be stated whether any investigation was conducted regarding multicollinearity among the variables.

**Author’s reply:**

Thanks for pointing out this methodological issue. It is indeed important to check if the independent variables are too strongly correlated. To test for multicollinearity in this study, we used variance inflation factors (VIF) and tolerance to examine the variables in the final multivariable linear regression models. A VIF value > 5 (tolerance<0.2) means a high and harmful level of correlation. Our results are listed on the table below. None of these independent variables have VIF values >5. We have added this point to the Methods (first paragraph, page 9) and Results sections (third paragraph, page 15).

**Table. Collinearity tests (variance inflation factors and tolerance) for the variables in in the multivariable models of early post-stroke cognitive performance.**

|  | **Tolerance** | **VIF** |
| --- | --- | --- |
| **Age (years)** | 0.889 | 1.12 |
| **Education (years)** | 0.854 | 1.17 |
| **NIHSS** | 0.516 | 1.94 |
| **GCS** | 0.504 | 1.99 |
| **ICH volume** | 0.832 | 1.20 |
| **MRI total CMBs** | 0.964 | 1.04 |

VIF = variance inflation factor (tolerance = 1/VIF)

5. The study states that patients were included within the first month after intracerebral hemorrhage and that cognitive assessment was performed "approximately one month later". However, the accepted time interval is not clearly stated. Similarly, it is not specified how many days after symptom onset the MRI scans were performed. Since both hematoma volume and some imaging findings may change over time, it is important to report the timing of imaging and cognitive assessment in more detail.

**Author’s reply:**

Thanks for your suggestion. Although we stated that acute ICH patients were prospectively recruited within one month of stroke onset, most of them were included within one week after onset. We have revised the text to state more clearly about the timing of CT (on the day upon arrival)/MRI scan (within 1 week after admission) and cognitive assessment (between the first and second month after stroke) (first paragraph, page 6).

6. As I previously mentioned regarding cognitive tests, there is missing data. For example, 92% of patients underwent MRI imaging, and 97% had APOE genotyping completed. How the missing data were handled in the analyses should be more clearly stated in the Methods section.

**Author’s reply:**

We thank you for this comment. For missing data, we adapted deletion methods (pairwise deletion), without imputation. In contrast to cognitive assessment, the percentage of missing data in patients who did not have MRI (8%) or APOE genotyping (3%) was very small. We have added the explicit statement for the strategy to hand missing data in Methods Section (first paragraph, page 9).

7. In the study, mostly only p-values are presented. The p-value alone tells me that a relationship exists, but it would be more useful to also see the magnitude and uncertainty of the effect. In multivariate regression analyses, it is recommended that regression coefficients, beta values, or other appropriate effect sizes be given along with 95% confidence intervals.

**Author’s reply:**

For the effect sizes with their uncertainty to be more informative, we have added 95% confidence intervals to all regression coefficients along with p-values in multivariate regression analyses in Table 3 and S3 Table. Thank you.

8. Only 25 patients were included in the probable CAA group. Although the authors generally stated the sample size as a limitation, the small size of the CAA subgroup in particular may have reduced the statistical power of the subgroup analyses. This point can be further emphasized in the Discussion section.

Author’s reply:

Yes, we agree with your opinion. In this study, we only conducted subgroup analysis to explore the associations of lobar and non-lobar CMB burden with early global cognitive function in patients with ICH. The small sample size of probable CAA group precluded us from other subgroup analysis as it may reduce the statistical power. We have further emphasized this point in the Disussion section (first paragraph, page 20)

9. The article is generally written in understandable English. However, a few minor spelling and grammatical errors are noticeable. For example, "generalized linear regression" is replaced with "linear," "MR angiography" with "angiography," and there is unnecessary word repetition in the phrase "associated clinical and neuroimaging associated factors" in the Conclusion section. Careful language checking before publication is recommended.

**Author’s reply:**

Many thanks for spending your precious time reviewing our manuscript. We have read through the main text carefully, invited Dr. Neshika Samarasekera in University of Edinburgh (her native language is English) to help with English revision, and corrected a few spelling and grammatical errors.
