# Supplementary material for: Clinical and neuroimaging features, and associations with early post-stroke cognition in patients with intracerebral hemorrhage in Taiwan
Source: PLoS One. 2026 Sep 21;21(9):e0358545. doi: 10.1371/journal.pone.0358545 (PMC13592605; doi:10.1371/journal.pone.0358545)
Supplement: Peer review history

# Peer review: Decision Letter 0

**DOI:** 10.1371/journal.pone.0358545.r001

**Authors:** Jinglu Ai

20 Jul 2026

PLOS One

Dear Dr. Tsai,

Thank you for submitting your manuscript to PLOS ONE. After careful consideration, we feel that it has merit but does not fully meet PLOS ONE’s publication criteria as it currently stands. Therefore, we invite you to submit a revised version of the manuscript that addresses the points raised during the review process.

The reviewers recognize the strengths of your study, including its prospective design, comprehensive neuroimaging evaluation, genetic analyses, and the clinical relevance of investigating factors associated with early cognitive outcomes following intracerebral hemorrhage. Both reviewers agree that the study is technically sound, the analyses are generally appropriate, and the conclusions are supported by the presented data. Accordingly, I believe the manuscript has merit and can be considered for publication after **minor revision** .

In preparing your revised manuscript, please address each reviewer comment carefully and provide a detailed point-by-point response. In particular, I encourage you to focus on the following issues:

Overall, the reviewers' comments are constructive and largely concern clarification, interpretation, and reporting rather than substantial revisions to the study itself. I look forward to receiving your revised manuscript and a detailed response addressing each point raised by the reviewers.

Please submit your revised manuscript by Aug 19 2026 11:59PM. If you will need more time than this to complete your revisions, please reply to this message or contact the journal office at plosone@plos.org. When you're ready to submit your revision, log on to https://www.editorialmanager.com/pone/ and select the 'Submissions Needing Revision' folder to locate your manuscript file.

- A letter that responds to each point raised by the academic editor and reviewer(s). You should upload this letter as a separate file labeled 'Response to Reviewers'.
- A marked-up copy of your manuscript that highlights changes made to the original version. You should upload this as a separate file labeled 'Revised Manuscript with Track Changes'.
- An unmarked version of your revised paper without tracked changes. You should upload this as a separate file labeled 'Manuscript'.

If you would like to make changes to your financial disclosure, please include your updated statement in your cover letter. Guidelines for resubmitting your figure files are available below the reviewer comments at the end of this letter.

If applicable, we recommend that you deposit your laboratory protocols in protocols.io to enhance the reproducibility of your results. Protocols.io assigns your protocol its own identifier (DOI) so that it can be cited independently in the future. For instructions see: https://journals.plos.org/plosone/s/submission-guidelines#loc-laboratory-protocols. Additionally, PLOS ONE offers an option for publishing peer-reviewed Lab Protocol articles, which describe protocols hosted on protocols.io. Read more information on sharing protocols at https://plos.org/protocols?utm_medium=editorial-email&utm_source=authorletters&utm_campaign=protocols.

As the corresponding author, your ORCID iD is verified in the submission system and will appear in the published article. PLOS supports the use of ORCID, and we encourage all coauthors to register for an ORCID iD and use it as well. Please encourage your coauthors to verify their ORCID iD within the submission system before final acceptance, as unverified ORCID iDs will not appear in the published article. *Only*  the individual author can complete the verification step; PLOS staff *cannot*  verify ORCID iDs on behalf of authors.

We look forward to receiving your revised manuscript.

Kind regards,

Jinglu Ai, M.D., Ph.D.

Academic Editor

PLOS One

Journal Requirements:

When submitting your revision, we need you to address these additional requirements.

1. Please ensure that your manuscript meets PLOS ONE's style requirements, including those for file naming. The PLOS ONE style templates can be found at

https://journals.plos.org/plosone/s/file?id=wjVg/PLOSOne_formatting_sample_main_body.pdf and

https://journals.plos.org/plosone/s/file?id=ba62/PLOSOne_formatting_sample_title_authors_affiliations.pdf

2. Thank you for stating the following in the Acknowledgments Section of your manuscript:

“This work was partly supported by the Cardinal Tien Hospital Research Grants (CTH 113A-2213, CTH 114A-2212; C.-F. Tsai). The funder had no role in the study design, data collection and analysis, decision to publish, or preparation of the manuscript”

We note that you have provided funding information that is not currently declared in your Funding Statement. However, funding information should not appear in the Acknowledgments section or other areas of your manuscript. We will only publish funding information present in the Funding Statement section of the online submission form.

Please remove any funding-related text from the manuscript and let us know how you would like to update your Funding Statement. Currently, your Funding Statement reads as follows:

“This work was partly supported by the Cardinal Tien Hospital Research Grants (CTH 113A-2213, CTH 114A-2212; C.-F. Tsai). The funder had no role in the study design, data collection and analysis, decision to publish, or preparation of the manuscript.”

Please include your amended statements within your cover letter; we will change the online submission form on your behalf.

3. Thank you for stating in your Funding Statement:

“This work was partly supported by the Cardinal Tien Hospital Research Grants (CTH 113A-2213, CTH 114A-2212; C.-F. Tsai). The funder had no role in the study design, data collection and analysis, decision to publish, or preparation of the manuscript.”

Please provide an amended statement that declares *all* the funding or sources of support (whether external or internal to your organization) received during this study, as detailed online in our guide for authors at http://journals.plos.org/plosone/s/submit-now.  Please also include the statement “There was no additional external funding received for this study.” in your updated Funding Statement.

Please include your amended Funding Statement within your cover letter. We will change the online submission form on your behalf.

4. Thank you for stating the following financial disclosure:

“This work was partly supported by the Cardinal Tien Hospital Research Grants (CTH 113A-2213, CTH 114A-2212; C.-F. Tsai). The funder had no role in the study design, data collection and analysis, decision to publish, or preparation of the manuscript.”

We note that one or more of the authors is affiliated with the funding organization, indicating the funder may have had some role in the design, data collection, analysis or preparation of your manuscript for publication; in other words, the funder played an indirect role through the participation of the co-authors. If the funding organization did not play a role in the study design, data collection and analysis, decision to publish, or preparation of the manuscript and only provided financial support in the form of authors' salaries and/or research materials, please do the following:

1. Review your statements relating to the author contributions, and ensure you have specifically and accurately indicated the role(s) that these authors had in your study. These amendments should be made in the online form.

2. Confirm in your cover letter that you agree with the following statement, and we will change the online submission form on your behalf:

“The funder provided support in the form of salaries for authors [insert relevant initials], but did not have any additional role in the study design, data collection and analysis, decision to publish, or preparation of the manuscript. The specific roles of these authors are articulated in the ‘author contributions’ section.

5. In the online submission form you indicate that your data is not available for proprietary reasons and have provided a contact point for accessing this data. Please note that your current contact point is a co-author on this manuscript. According to our Data Policy, the contact point must not be an author on the manuscript and must be an institutional contact, ideally not an individual. Please revise your data statement to a non-author institutional point of contact, such as a data access or ethics committee, and send this to us via return email. Please also include contact information for the third party organization, and please include the full citation of where the data can be found.

6. Your ethics statement should only appear in the Methods section of your manuscript. If your ethics statement is written in any section besides the Methods, please move it to the Methods section and delete it from any other section. Please ensure that your ethics statement is included in your manuscript, as the ethics statement entered into the online submission form will not be published alongside your manuscript.

7. If the reviewer comments include a recommendation to cite specific previously published works, please review and evaluate these publications to determine whether they are relevant and should be cited. There is no requirement to cite these works unless the editor has indicated otherwise.

8. Please review your reference list to ensure that it is complete and correct. If you have cited papers that have been retracted, please include the rationale for doing so in the manuscript text, or remove these references and replace them with relevant current references. Any changes to the reference list should be mentioned in the rebuttal letter that accompanies your revised manuscript. If you need to cite a retracted article, indicate the article’s retracted status in the References list and also include a citation and full reference for the retraction notice.

[Note: HTML markup is below. Please do not edit.]

Reviewers' comments:

Reviewer's Responses to Questions

**Comments to the Author**

1. Is the manuscript technically sound, and do the data support the conclusions?

Reviewer #1: Yes

Reviewer #2: Yes

**********

2. Has the statistical analysis been performed appropriately and rigorously?

Reviewer #1: Yes

Reviewer #2: Yes

**********

3. Have the authors made all data underlying the findings in their manuscript fully available?

The PLOS Data policy

Reviewer #1: Yes

Reviewer #2: Yes

**********

4. Is the manuscript presented in an intelligible fashion and written in standard English?

Reviewer #1: Yes

Reviewer #2: Yes

**********

Reviewer #1: The authors present the results of a prospective, hospital-based study from two large hospitals in Taiwan aimed to make comparisons between different intracerebral hemorrhage (ICH) locations and etiologies, and to investigate the factors associated with early poststroke cognition. A total of 202 acute spontaneous ICH patients were included in the study. The authors demonstrated differences in clinical and imaging features, major risk factors, and early post-stroke cognitive function between different locations and etiologies. Age, education, stroke severity, ICH volume and lobar CMB burden were the associated factors for early post-stroke cognition. The study is potentially interesting, but can be improved if the following considerations are addressed:

1.The text should place greater emphasis on the fact that acute lobar cerebral hemorrhages present a different clinical profile and a more severe early prognosis than deep subcortical intracerebral hemorrhages and should add that non-hypertensive mechanisms of intracerebral hemorrhages predominate in the lobar location. Please compare their findings with data from a study reported in Biomedicines 2023 Jan 16;11(1):223.

2.The authors have certainly and appropriately mentioned in the text the clinical severity of intracerebral hemorrhages. However, it would be interesting to mention also that there is a subgroup of hypertensive cerebral hemorrhages of small size of the lesion, which clinically manifest as a lacunar syndrome (“hemorrhagic pure motor stroke”). This subgroup accounts for 3.3% of cerebral hemorrhages and are associated with a more favorable prognosis as compared to the remaining group of intracerebral hemorrhages, usually without in-hospital deaths and symptom-free at discharge in 25% of cases, as described in a clinical series of this condition (see and include this supporting reference: Eur J Neurol. 2007 Feb;14(2):219-23. doi: 10.1111/j.1468-1331.2006.01660.x. PMID: 17250733.). Did the authors consider this in their study?

3.It would be interesting to add the results in the subgroup of very old patients (aged 85 years or older) given that demographics, risk factors and prognosis are quite different in this age group of stroke patients (Evidence supporting this point should be included after searching in PubMed for suitable publications). Did the authors consider this in their study?

4.A brief concluding comment on other possible lines of future research on the presented topic would be appreciated.

Reviewer #2: This study investigated the clinical characteristics, risk factors, radiological findings, and early cognitive performance of patients diagnosed with intracerebral hemorrhage in Taiwan, according to the localization of the hemorrhage. It is a prospective, double-center observational study. The strengths of the study are its prospective nature, the inclusion of genetic analyses, and the combined evaluation of neuroimaging findings. Overall, the study design, the tests used, and the statistical methods applied are appropriate. The data are consistent with the results obtained. However, I believe that the methodology needs to be made more transparent and the interpretation of some results needs to be strengthened. I would like to state that fulfilling the following suggestions will increase the scientific validity of this study and will be beneficial for readers to better understand it.

1. A total of 202 patients were included in the study, but it was found that MMSE and MoCA tests could only be performed on 134 patients and 119 patients, respectively. The authors stated that part of the reason for this was hemodynamic instability, impaired consciousness, and early mortality. However, the clinical characteristics (age, NIHSS, GCS, ICH volume, mortality, etc.) of patients who could not be included in the cognitive assessment were not specified, and they were not compared with those of other patients. It is highly probable that patients who could not participate in the cognitive assessment had a more severe stroke. Therefore, a systematic selection bias may have occurred in the cognitive analyses, and the results obtained may underestimate the actual frequency of cognitive impairment. The authors could briefly present the characteristics of patients who could not undergo cognitive assessment, or at least discuss the possible impact of this on the results in more detail in the Discussion section. I believe this is important to strengthen the study.

2. The phrase "post-stroke cognition" is frequently used in many parts of the article, including the title and aims. However, cognitive assessment was performed only with MMSE and MoCA. While these two tests are widely used, valid, and reliable tools for global cognitive screening, they cannot comprehensively assess executive functions, attention, processing speed, working memory, and other specific cognitive domains that are frequently affected, especially after intracerebral hemorrhage. Therefore, these tests alone do not provide comprehensive information regarding cognitive assessment. For this reason, it would be more appropriate to interpret the results in the context of early global cognitive performance measured by MMSE and MoCA, rather than "post-stroke cognition." It is recommended that this limitation be emphasized more clearly in the Discussion and Conclusion sections.

3. The authors state that they used a generalized linear regression model for multivariate analysis. In general, this appears to be a suitable method. However, if the approach by which the variables were included in the model (clinical significance, literature information, or univariate analysis results) were explained more clearly, and the basic characteristics of the model were briefly stated, the methodology and analysis of the study would be stronger and more transparent. 4. Some variables included in the multivariate model (e.g., GCS, NIHSS, and ICH volume) may be considered clinically related to each other. If evaluated, it should be stated whether any investigation was conducted regarding multicollinearity among the variables.

5. The study states that patients were included within the first month after intracerebral hemorrhage and that cognitive assessment was performed "approximately one month later". However, the accepted time interval is not clearly stated. Similarly, it is not specified how many days after symptom onset the MRI scans were performed. Since both hematoma volume and some imaging findings may change over time, it is important to report the timing of imaging and cognitive assessment in more detail.

6. As I previously mentioned regarding cognitive tests, there is missing data. For example, 92% of patients underwent MRI imaging, and 97% had APOE genotyping completed. How the missing data were handled in the analyses should be more clearly stated in the methods section.

7. In the study, mostly only p-values are presented. The p-value alone tells me that a relationship exists, but it would be more useful to also see the magnitude and uncertainty of the effect. In multivariate regression analyses, it is recommended that regression coefficients, beta values, or other appropriate effect sizes be given along with 95% confidence intervals.

8. Only 25 patients were included in the probable CAA group. Although the authors generally stated the sample size as a limitation, the small size of the CAA subgroup in particular may have reduced the statistical power of the subgroup analyses. This point can be further emphasized in the Discussion section.

9. The article is generally written in understandable English. However, a few minor spelling and grammatical errors are noticeable. For example, "generalized linear regression" is replaced with "linear," "MR angiography" with "angiography," and there is unnecessary word repetition in the phrase "associated clinical and neuroimaging associated factors" in the Conclusion section. Careful language checking before publication is recommended.

In conclusion, the scientific question of the study is important, and the results obtained and the data presented are consistent. The suggestions I have mentioned are largely aimed at detailing the reporting, increasing methodological transparency, and interpreting the results more carefully. Therefore, I believe that publication of the study after minor revision would be appropriate.

**********

what does this mean?). If published, this will include your full peer review and any attached files.

If you choose “no”, your identity will remain anonymous but your review may still be made public.

**Do you want your identity to be public for this peer review?**  For information about this choice, including consent withdrawal, please see our Privacy Policy

Reviewer #1: **Yes:**  Adrià Arboix

Reviewer #2: No

**********

[NOTE: If reviewer comments were submitted as an attachment file, they will be attached to this email and accessible via the submission site. Please log into your account, locate the manuscript record, and check for the action link "View Attachments". If this link does not appear, there are no attachment files.]

To ensure your figures meet our technical requirements, please review our figure guidelines: https://journals.plos.org/plosone/s/figures

You may also use PLOS’s free figure tool, NAAS, to help you prepare publication quality figures: https://journals.plos.org/plosone/s/figures#loc-tools-for-figure-preparation.

NAAS will assess whether your figures meet our technical requirements by comparing each figure against our figure specifications.

# Author comment: Author response to Decision Letter 1

**DOI:** 10.1371/journal.pone.0358545.r002

20 Aug 2026

Dear Academic Editor Jinglu Ai and Reviewers,

Many thanks for your positive feedback and for giving us the opportunity to revise our manuscript. We had made corresponding changes based on PLOS ONE's style requirements, Editor and reviewer’s suggestions, and prepared a letter “Response to Reviewers” below, replying to Reviewers’ comments point by point and stating the page and paragraph number of specific changes in the revised manuscript. Also, we have updated our Funding Statement and Data Availability Statement in the cover letter.

Journal Requirements:

1. Please ensure that your manuscript meets PLOS ONE's style requirements, including those for file naming. The PLOS ONE style templates can be found at

https://journals.plos.org/plosone/s/file?id=wjVg/PLOSOne_formatting_sample_main_body.pdf and

https://journals.plos.org/plosone/s/file?id=ba62/PLOSOne_formatting_sample_title_authors_affiliations.pdf

Author’s reply:

Thanks for your reminder. We have checked journal formatting guidelines to meet PLOS ONE’s style.

2. Thank you for stating the following in the Acknowledgments Section of your manuscript:

“This work was partly supported by the Cardinal Tien Hospital Research Grants (CTH 113A-2213, CTH 114A-2212; C.-F. Tsai). The funder had no role in the study design, data collection and analysis, decision to publish, or preparation of the manuscript”

We note that you have provided funding information that is not currently declared in your Funding Statement. However, funding information should not appear in the Acknowledgments section or other areas of your manuscript. We will only publish funding information present in the Funding Statement section of the online submission form.

Please remove any funding-related text from the manuscript and let us know how you would like to update your Funding Statement. Currently, your Funding Statement reads as follows:

“This work was partly supported by the Cardinal Tien Hospital Research Grants (CTH 113A-2213, CTH 114A-2212; C.-F. Tsai). The funder had no role in the study design, data collection and analysis, decision to publish, or preparation of the manuscript.”

Please include your amended statements within your cover letter; we will change the online submission form on your behalf.

Author’s reply:

We have removed any funding-related text from the manuscript, updated our Funding Statement below, and included the amended statement in our cover letter.

3. Thank you for stating in your Funding Statement:

“This work was partly supported by the Cardinal Tien Hospital Research Grants (CTH 113A-2213, CTH 114A-2212; C.-F. Tsai). The funder had no role in the study design, data collection and analysis, decision to publish, or preparation of the manuscript.”

Please provide an amended statement that declares *all* the funding or sources of support (whether external or internal to your organization) received during this study, as detailed online in our guide for authors at http://journals.plos.org/plosone/s/submit-now. Please also include the statement “There was no additional external funding received for this study.” in your updated Funding Statement.

Please include your amended Funding Statement within your cover letter. We will change the online submission form on your behalf.

Author’s reply:

Thanks for your suggestion. We have updated the Funding Statement: “This work was partly supported by the Cardinal Tien Hospital Research Grants (CTH 113A-2213, CTH 114A-2212; C.-F. Tsai). There was no additional external funding received for this study. All the funding or sources of support received during this study had no role in study design, data collection and analysis, decision to publish, or preparation of the manuscript.” Also, we have included the amended Funding Statement in our cover letter.

4. Thank you for stating the following financial disclosure:

“This work was partly supported by the Cardinal Tien Hospital Research Grants (CTH 113A-2213, CTH 114A-2212; C.-F. Tsai). The funder had no role in the study design, data collection and analysis, decision to publish, or preparation of the manuscript.”

We note that one or more of the authors is affiliated with the funding organization, indicating the funder may have had some role in the design, data collection, analysis or preparation of your manuscript for publication; in other words, the funder played an indirect role through the participation of the co-authors. If the funding organization did not play a role in the study design, data collection and analysis, decision to publish, or preparation of the manuscript and only provided financial support in the form of authors' salaries and/or research materials, please do the following:

1. Review your statements relating to the author contributions, and ensure you have specifically and accurately indicated the role(s) that these authors had in your study. These amendments should be made in the online form.

2. Confirm in your cover letter that you agree with the following statement, and we will change the online submission form on your behalf:

“The funder provided support in the form of salaries for authors [insert relevant initials], but did not have any additional role in the study design, data collection and analysis, decision to publish, or preparation of the manuscript. The specific roles of these authors are articulated in the ‘author contributions’ section.

Author’s reply:

Yes, we have reviewed the statement related to the author contributions and have confirmed in our cover letter that we agree with the following statement: “The funder provided support in the form of salaries for authors [CT, YW, and PY], but did not have any additional role in the study design, data collection and analysis, decision to publish, or preparation of the manuscript. The specific roles of these authors are articulated in the ‘author contributions’ section".

5. In the online submission form you indicate that your data is not available for proprietary reasons and have provided a contact point for accessing this data. Please note that your current contact point is a co-author on this manuscript. According to our Data Policy, the contact point must not be an author on the manuscript and must be an institutional contact, ideally not an individual. Please revise your data statement to a non-author institutional point of contact, such as a data access or ethics committee, and send this to us via return email. Please also include contact information for the third party organization, and please include the full citation of where the data can be found.

Author’s reply:

We have revised our Data Availability Statement in the cover letter as follows - Data generated or analyzed during this study are included in the manuscript and its Supporting Information files. Because of ethical and privacy consideration, further enquiries for other relevant data can be made to Institution Review Board of the Cardinal Tien Hospital (https://www.cth.org.tw/?aid=52&pid=70) (irb.cth@gmail.com) for researchers who meet the criteria for access to confidential data.

6. Your ethics statement should only appear in the Methods section of your manuscript. If your ethics statement is written in any section besides the Methods, please move it to the Methods section and delete it from any other section. Please ensure that your ethics statement is included in your manuscript, as the ethics statement entered into the online submission form will not be published alongside your manuscript.

Authors’ reply:

Thank you. We have placed ethic statement in the Methods section and removed it from any other section (first paragraph, page 6).

7. If the reviewer comments include a recommendation to cite specific previously published works, please review and evaluate these publications to determine whether they are relevant and should be cited. There is no requirement to cite these works unless the editor has indicated otherwise.

Author’s reply:

Thanks for your reminder. We have reviewed and evaluated these publications carefully and only cited the paper which is relevant to this study (Reference 24).

8. Please review your reference list to ensure that it is complete and correct. If you have cited papers that have been retracted, please include the rationale for doing so in the manuscript text, or remove these references and replace them with relevant current references. Any changes to the reference list should be mentioned in the rebuttal letter that accompanies your revised manuscript. If you need to cite a retracted article, indicate the article’s retracted status in the References list and also include a citation and full reference for the retraction notice.

Author’s reply:

We have reviewed our references, marked the added or changed references in the “Response to Reviewers” and our revised manuscript. Thank you.

Reviewers’ comments

Reviewer #1: The authors present the results of a prospective, hospital-based study from two large hospitals in Taiwan aimed to make comparisons between different intracerebral hemorrhage (ICH) locations and etiologies, and to investigate the factors associated with early poststroke cognition. A total of 202 acute spontaneous ICH patients were included in the study. The authors demonstrated differences in clinical and imaging features, major risk factors, and early post-stroke cognitive function between different locations and etiologies. Age, education, stroke severity, ICH volume and lobar CMB burden were the associated factors for early post-stroke cognition. The study is potentially interesting, but can be improved if the following considerations are addressed:

1.The text should place greater emphasis on the fact that acute lobar cerebral hemorrhages present a different clinical profile and a more severe early prognosis than deep subcortical intracerebral hemorrhages and should add that non-hypertensive mechanisms of intracerebral hemorrhages predominate in the lobar location. Please compare their findings with data from a study reported in Biomedicines 2023 Jan 16;11(1):223.

Author’s reply:

Many thanks for your valuable suggestions. We have revised our manuscript to put greater emphasis on the fact that patients with lobar cerebral hemorrhages have a different clinical profile and a more severe early prognosis with a higher 1-month case-fatality than those with deep intracerebral hemorrhages (first paragraph, page 18). Also, we made comparisons with findings in another ICH study mentioned above and added it in our references (Biomedicines 2023 Jan 16;11(1):223, Reference 24). In addition, we have added the statement that 61% of patients with lobar ICH fulfill the diagnosis of probable CAA and is the predominate mechanism (first paragraph, page 18).

2.The authors have certainly and appropriately mentioned in the text the clinical severity of intracerebral hemorrhages. However, it would be interesting to mention also that there is a subgroup of hypertensive cerebral hemorrhages of small size of the lesion, which clinically manifest as a lacunar syndrome (“hemorrhagic pure motor stroke”). This subgroup accounts for 3.3% of cerebral hemorrhages and are associated with a more favorable prognosis as compared to the remaining group of intracerebral hemorrhages, usually without in-hospital deaths and symptom-free at discharge in 25% of cases, as described in a clinical series of this condition (see and include this supporting reference: Eur J Neurol. 2007 Feb;14(2):219-23. doi: 10.1111/j.1468-1331.2006.01660.x. PMID: 17250733.). Did the authors consider this in their study?

Author’s reply:

Thanks for your question. That’s indeed interesting. However, after reading the paper published on 2007 Eur J Neurol and checking the records of our study, we did not find “hemorrhagic pure motor stroke” in our patients. It’s probably a very infrequent stroke subtype and our sample size is limited. We are glad to pay more attention to this point in the future.

3.It would be interesting to add the results in the subgroup of very old patients (aged 85 years or older) given that demographics, risk factors and prognosis are quite different in this age group of stroke patients (Evidence supporting this point should be included after searching in PubMed for suitable publications). Did the authors consider this in their study?

Author’s reply:

Thanks for your suggestion. We have managed to make a table to compare the very old patients (aged 85 years or older) versus others below. The very old patients with ICH have a significantly higher proportion of atrial fibrillation than others, while the distribution of sex, GCS, NIHSS, ICH volume, other vascular risk factors, and 1-month case fatality do not differ significantly between these two groups. However, there are only 4 very old patients (2%) in our study, which may reduce statistical power. The results should be interpreted with caution.

Table. Comparisons between the very old patients and other patients with ICH

<85 years

(N=198, 98%) ≥85 years

(N=4, 2%) P

Mean age (year) 60.0(±11.8) 89.5 (±7.7) <0.001*

Sex (male) (%) 130 (66%) 1 (25%) 0.126

GCS 13.8 (±2.4) 13.3 (± 2.2) 0.152

NIHSS 8.8 (± 7.9) 17.3 (±11.1) 0.107

ICH score 0.7 (±0.9) 1.5 (± 1.0) 0.069

1m case fatality 3 (2%) 0 (0%) 1.000

Hypertension(N) (%) 189 (96%) 4 (100%) 1.000

Diabetes (N) (%) 47 (24%) 1 (25%) 1.000

Atrial fibrillation (N) (%) 9 (5%) 2 (50%) 0.015*

Ischemic heart(N) (%) 20 (10%) 0 (0%) 1.000

Hypercholesterolemia(N) (%) 56 (28%) 0 (0%) 0.577

Hypertriglycemia (N) (%) 13 (7%) 0 (0%) 1.000

Smoking (N) (%) 105 (53%) 1 (25%) 0.348

Alcohol (N) (%) 46 (23%) 1 (25%) 1.000

Previous stroke(N) (%) 30 (15%) 1 (25%) 0.489

Pre-ICH antiplatelet(N) (%) 24 (12%) 0 (0%) 1.000

Pre-ICH anticoagulation(N) (%) 5 (3%) 1 (25%) 0.114

Family history of stroke (N) (%) 57 (29%) 1 (25%) 1.000

ICH volume (ml) 23 (±29) 31 (±30) 0.567

4.A brief concluding comment on other possible lines of future research on the presented topic would be appreciated.

Author’s reply:

We are grateful for your comments. We have added the possible directions of further research in the Conclusion Section (first paragraph, page 21).

Reviewer #2: This study investigated the clinical characteristics, risk factors, radiological findings, and early cognitive performance of patients diagnosed with intracerebral hemorrhage in Taiwan, according to the localization of the hemorrhage. It is a prospective, double-center observational study. The strengths of the study are its prospective nature, the inclusion of genetic analyses, and the combined evaluation of neuroimaging findings. Overall, the study design, the tests used, and the statistical methods applied are appropriate. The data are consistent with the results obtained. However, I believe that the methodology needs to be made more transparent and the interpretation of some results needs to be strengthened. I would like to state that fulfilling the following suggestions will increase the scientific validity of this study and will be beneficial for readers to better understand it.

1. A total of 202 patients were included in the study, but it was found that MMSE and MoCA tests could only be performed on 134 patients and 119 patients, respectively. The authors stated that part of the reason for this was hemodynamic instability, impaired consciousness, and early mortality. However, the clinical characteristics (age, NIHSS, GCS, ICH volume, mortality, etc.) of patients who could not be included in the cognitive assessment were not specified, and they were not compared with those of other patients. It is highly probable that patients who could not participate in the cognitive assessment had a more severe stroke. Therefore, a systematic selection bias may have occurred in the cognitive analyses, and the results obtained may underestimate the actual frequency of cognitive impairment. The authors could briefly present the characteristics of patients who could not undergo cognitive assessment, or at least discuss the possible impact of this on the results in more detail in the Discussion section. I believe this is important to strengthen the study.

Author’s reply:

Many thanks for your intelligent insight and suggestion. We agree with your opinion that patients who co

Attachment Submitted filename: 2026 PLOS ONE revision - Response to Reviewers.docx

# Editor report: Decision Letter 1

**DOI:** 10.1371/journal.pone.0358545.r003

**Authors:** Jinglu Ai

3 Sep 2026

Clinical and neuroimaging features, and associations with early post-stroke cognition in patients with intracerebral hemorrhage in Taiwan

PONE-D-26-33405R1

Dear Dr. Tsai,

We’re pleased to inform you that your manuscript has been judged scientifically suitable for publication and will be formally accepted for publication once it meets all outstanding technical requirements.

Within one week, you’ll receive an e-mail detailing the required amendments. When these have been addressed, you’ll receive a formal acceptance letter and your manuscript will be scheduled for publication.

An invoice will be generated when your article is formally accepted. Please note, if your institution has a publishing partnership with PLOS and your article meets the relevant criteria, all or part of your publication costs will be covered. Please make sure your user information is up-to-date by logging into Editorial Manager at Editorial Manager® and clicking the ‘Update My Information' link at the top of the page. For questions related to billing, please contact billing support.

If your institution or institutions have a press office, please notify them about your upcoming paper to help maximize its impact. If they’ll be preparing press materials, please inform our press team as soon as possible -- no later than 48 hours after receiving the formal acceptance. Your manuscript will remain under strict press embargo until 2 pm Eastern Time on the date of publication. For more information, please contact onepress@plos.org.

Kind regards,

Jinglu Ai, M.D., Ph.D.

Academic Editor

PLOS One

Additional Editor Comments (optional):

Reviewers' comments:

# Editor report: Acceptance letter

**DOI:** 10.1371/journal.pone.0358545.r004

**Authors:** Jinglu Ai

PONE-D-26-33405R1

PLOS One

Dear Dr. Tsai,

I'm pleased to inform you that your manuscript has been deemed suitable for publication in PLOS One. Congratulations! Your manuscript is now being handed over to our production team.

At this stage, our production department will prepare your paper for publication. This includes ensuring the following:

* All references, tables, and figures are properly cited

* All relevant supporting information is included in the manuscript submission,

* There are no issues that prevent the paper from being properly typeset

You will receive further instructions from the production team, including instructions on how to review your proof when it is ready. Please keep in mind that we are working through a large volume of accepted articles, so please give us a few days to review your paper and let you know the next and final steps.

Lastly, if your institution or institutions have a press office, please let them know about your upcoming paper now to help maximize its impact. If they'll be preparing press materials, please inform our press team within the next 48 hours. Your manuscript will remain under strict press embargo until 2 pm Eastern Time on the date of publication. For more information, please contact onepress@plos.org.

You will receive an invoice from PLOS for your publication fee after your manuscript has reached the completed accept phase. If you receive an email requesting payment before acceptance or for any other service, this may be a phishing scheme. Learn how to identify phishing emails and protect your accounts at https://explore.plos.org/phishing.

If we can help with anything else, please email us at customercare@plos.org.

Thank you for submitting your work to PLOS One and supporting open access.

Kind regards,

PLOS One Editorial Office Staff

on behalf of

Dr. Jinglu Ai

Academic Editor

PLOS One
